# Turning sound and force into light with AlN:Mn^2+^ mechanoluminescence

**DOI:** 10.1126/sciadv.aed5469

**Published:** 2026-06-26

**Authors:** Justyna Barzowska, Syed Shabhi Haider, Teng Zheng, Andrzej Suchocki, Philippe F. Smet, Marcin Runowski, Nerine J. Cherepy, Dengfeng Peng, Sebastian Mahlik

**Affiliations:** ^1^ Institute of Experimental Physics, Faculty of Mathematics, Physics and Informatics, University of Gdansk, Wita Stwosza 57, 80-308 Gdansk, Poland.; ^2^ Institute of Physics, Polish Academy of Sciences, Al. Lotników 32/46, Warsaw 02-668, Poland.; ^3^ Department of Mechanical Engineering, College of Engineering & Mines, University of North Dakota, Grand Forks, ND 58202, USA.; ^4^ School of Information and Electrical Engineering, Hangzhou City University, Hangzhou 310015, China.; ^5^ Department of Solid State Sciences, LumiLab, Ghent University, Krijgslaan 285-S1, 9000 Ghent, Belgium.; ^6^ Faculty of Chemistry, Adam Mickiewicz University, Uniwersytetu Poznańskiego 8, 61-614 Poznań, Poland.; ^7^ Lawrence Livermore National Laboratory, Livermore, CA 94550, USA.; ^8^ Shenzhen Key Laboratory of Intelligent Optical Measurement and Detection, Shenzhen University, Shenzhen 518060, China.; ^9^ Key Laboratory of Optoelectronic Devices and Systems of Ministry of Education and Guangdong Province, College of Physics and Optoelectronic Engineering, Shenzhen University, Shenzhen 518060, China.; ^10^ State Key Laboratory of Radio Frequency Heterogeneous Integration, Shenzhen University, Shenzhen 518060, China.

## Abstract

The development of multifunctional photo- and mechanoluminescent materials capable of responding to diverse external stimuli, enabling the visualization of mechanical action and acoustic waves, is essential for next-generation sensing and optoelectronic technologies. In this study, we present Mn^2+^-doped aluminum nitride (AlN:Mn^2+^) as a luminescent material that simultaneously exhibits bright orange photoluminescence, strong and long-lasting persistent luminescence, thermoluminescence, and mechanoluminescence (ML). AlN:Mn^2+^ demonstrates multimodal ML behavior under various mechanical stimuli, including friction, impact, and ultrasonic excitation, highlighting its potential for force and sound sensing applications across a wide range of environments. This is crucial for next-generation energy transformation, enabling the conversion of heat, force, and sound into light. The integration of persistent, thermal, mechanical, and acoustic luminescence within a single, robust, and chemically stable material makes AlN:Mn^2+^ a promising candidate for remote, adaptive optomechanical, and photoacoustic applications in biomedical diagnostics, structural health monitoring, anticounterfeiting, remote sensing, and innovative electronics.

## Teaser

Mn^2+^-doped aluminum nitride allows conversion of heat, force, and sound into light and acoustic or mechanical waves visualization.

## INTRODUCTION

Mechanoluminescence (ML) refers to the phenomenon of converting mechanical energy into light when a material is subjected to mechanical stress (*1*–*3*). Various mechanical stimuli can be applied as excitation sources, including compression, bending, stretching, impact deformation, cutting, friction, grinding, vibration, scratching, or even acoustic waves (*4*, *5*). Among these, acoustic waves represent a form of remote excitation, efficiently transferring energy and propagating as longitudinal or transverse waves through solids, liquids, or biological tissues via molecular vibrations (*6*, *7*). Although traditional ML induced by friction or impact holds substantial promise across various application fields, acoustic wave–triggered ML (e.g., via ultrasound) offers distinct and enhanced advantages: (i) real-time, noninvasive (remote) excitation that minimizes mechanical damage, making it ideal for fragile or precision materials; (ii) efficient energy transfer, enabling uniform excitation and higher luminescence efficiency; (iii) tunable control via ultrasonic (US) frequency and amplitude, enhancing adaptability across diverse applications; and (iv) deep penetration capability exceeding 10 cm in biological tissues and allowing ML activation in metals, ceramics, composites, and soft tissues (*8*, *9*). In addition, this approach provides high spatiotemporal resolution, biosafety, and low-cost implementation. Thus, sound-induced ML is a promising tool for noninvasive bioimaging, targeted therapy, sensing, optogenetics, and photonic interfaces (*10*–*12*). However, research in this area is still in its early stages and requires further development.

AlN, as a key member of the III-nitride semiconductor family, plays a critical role in advancing modern electronic and optoelectronic technologies (*13*–*15*). Beyond its well-established roles in electronics and semiconductor devices, AlN is also used in blue-violet light-emitting diodes (LEDs), short-wavelength lasers, and ultraviolet (UV) detectors (*16*, *17*). The luminescence properties of AlN arise from various intrinsic and extrinsic defects within its wide bandgap, which act as emission centers across UV to visible wavelengths. These defect-related emissions are highly responsive to external stimuli. AlN:Mn^2+^ is distinguished by its excellent chemical and moisture stability, as well as its thermal durability, mechanical robustness, and oxidation resistance (*18*, *19*). These attributes enhance the durability of the material in harsh environments and support its long-term performance. The substitution of Mn^2+^ for Al^3+^ introduces prominent defect complexes into the wurtzite structure of AlN, which may enhance and prolong the ML phenomenon, and allow the optical properties to be modulated by external stimuli such as temperature, pressure, and mechanical force (*2*, *12*, *20*). In addition, the unique abundance and distribution of trap states in AlN:Mn^2+^ endow it with an exceptional capacity to store excitation energy, which is later released as intense and persistent luminescence (PersL).

In this work, we report that the wide-bandgap semiconductor AlN doped with Mn^2+^ is a multifunctional luminescent material that simultaneously exhibits bright orange photoluminescence (PL), strong and long-lasting PersL, thermoluminescence (TL), and ML induced with different stimuli, including friction, impact, stretching, and sound (acoustic excitation). Notably, AlN:Mn^2+^ exhibits sound-induced ML, highlighting its potential for visualizing acoustic waves. Furthermore, the material functions as a highly sensitive, PL-based multimodal temperature sensor, enabling advanced thermometry through band ratio, centroid, and bandwidth analysis. This integration of multistimuli optical responses positions AlN:Mn^2+^ as a next-generation platform for remote, adaptive, and light-responsive technologies in emerging fields such as biomedical diagnostics, sound/force visualization, advanced anticounterfeiting, structural health monitoring, and innovative industrial systems.

## RESULTS

### Basic structural properties

**Fig. 1.**
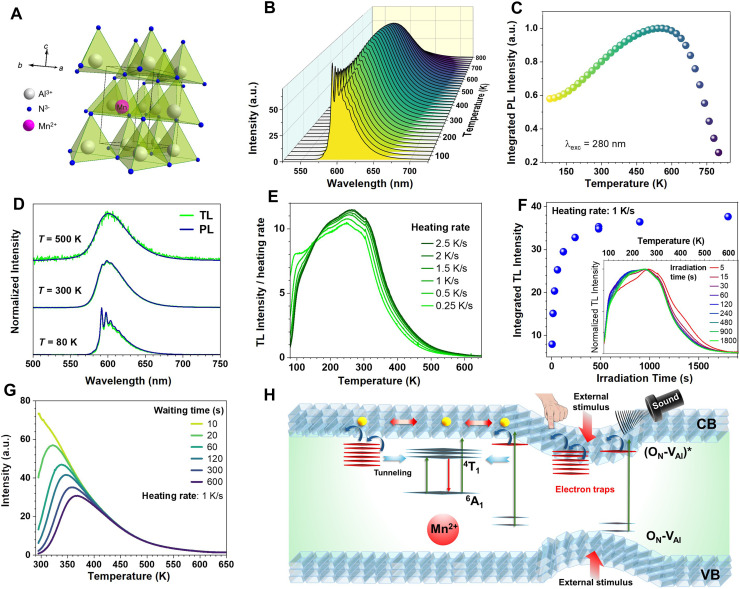
PL and TL of the AlN:Mn^2+^ material. (**A**) 3D atomic arrangement in the Mn^2+^-doped wurtzite AlN crystal. (**B**) Temperature-dependent PL spectra of the AlN:Mn^2+^, excited at 280 nm. (**C**) Temperature dependence of the integrated PL intensity (λ_ex_ = 280 nm). (**D**) Comparison of normalized PL (blue) and TL (green) spectra measured at 80, 300, and 500 K. (**E**) TL glow curves measured at different heating rates. Before each TL experiment, the sample was irradiated for 2 min with 280 nm from an LED and then kept in the dark for 1 min. (**F**) The dependence of total TL intensity on UV irradiation time; the inset depicts normalized TL glow curves for different UV irradiation times. (**G**) TL glow curves measured at different waiting times after the irradiation stage (performed at RT). (**H**) Scheme of energy level diagram of AlN:Mn^2+^.

The details of the synthesis and physicochemical characterization (figs. S1 to S6) of the AlN:Mn^2+^ powder are shown in the Supplementary Materials. Note, in this work, we used the aluminum nitride material doped with a very low concentration of Mn^2+^ of 0.06 mole % (mol %), which exhibits the best luminescence performance (highest signal intensity), compared to the samples with lower or higher Mn^2+^ concentrations, as discussed in detail in the Supplementary Materials and in our previous work (*21*). Figure S1 depicts the x-ray diffraction (XRD) pattern of the tested sample alongside the wurtzite AlN standard PDF 01-086-4277. The XRD pattern perfectly matches the standard wurtzite AlN reference (PDF 01-086-4277, P63mc), verifying that the crystal structure is a stable, pure-phase material. The three-dimensional (3D) atomic arrangement in Mn^2+^-doped wurtzite AlN crystals is illustrated in Fig. 1A, showing the characteristic hexagonal structure of the unit cell. Please note that Mn^2+^ substitution for Al^3+^ introduces pronounced defect complexes in the wurtzite structure, due to the larger ionic size of Mn^2+^ ($R_{\text{Mn}}^{2+}$ = 0.66 Å; $R_{\text{Al}}^{3+}$ = 0.39 Å) as well as the necessary charge compensation. In particular, the formation of Mn^2+^-V_Al_ defect complexes is one of the possible, yet highly probable, mechanisms, as it is energetically favored and could contribute to the stabilization of the structure by locally compensating for the charge imbalance and minimizing lattice strain caused by the size mismatch between the Mn^2+^ and Al^3+^ ions. Moreover, AlN may contain a variety of intrinsic and extrinsic defects, intentional and nonintentional, including their complexes. Typically, vacancies on both cation and anion sublattices, interstitials, antisites (N_Al_ and Al_N_), nonintentional oxygen, which can be introduced during sample preparation, and many others are present (*21*–*23*).

These defects and defect complexes influence the electronic and trapping properties of the material; however, at the low Mn^2+^ concentration used in this study, they do not measurably perturb the average crystal lattice. Accordingly, no substantial changes in the vibrational response are detected by Raman spectroscopy. The Raman spectrum of AlN:Mn^2+^ exhibits four characteristic modes at 611, 656, 667, and 900 cm^−1^ (fig. S2), corresponding to the A_1_(TO), E_2_, E_1_(TO), and A_1_(LO)/E_1_(LO) modes of wurtzite AlN, respectively. Their positions and linewidths closely match those of undoped AlN (*24*). The morphological characteristics of the sample were investigated using scanning electron microscopy (SEM) at different magnifications, as shown in fig. S3. The SEM images revealed that the AlN: Mn^2+^ sample comprises particles with well-defined, sharp edges. The grains are irregularly shaped, with minimal evidence of rounding or smoothing at the edges. The particle surfaces appear smooth, with distinct hexagonal steps and visible faceting. The powder predominantly contains particles with sizes ranging from 3 to 10 μm, along with a fraction of finer particles and the largest agglomerates, which do not exceed 30 μm. The average particle size, calculated from the overview SEM image and presented in the histogram of particle size distribution (fig. S3B), is around 5.5 μm. Figure S4 shows an SEM image of the cross-sectional view of the sample sheet prepared for the ML experiments. The UV-transparent polypropylene (PP) adhesive tape was 20 μm thick, and the sample layer was approximately 30 μm thick. The sample powder is observed as the sharply focused region in the SEM image, clearly distinct from the adhesive tape.

### PL and TL properties

The characteristic Mn^2+^ emission, attributed to the spin-forbidden ^4^T_1_ → ^6^A_1_ transition, exhibits excellent sensitivity to the external temperature environment. To investigate the spectroscopic properties under thermal conditions, a series of AlN:Mn^2+^ PL spectra, excited at 280 nm and measured at temperatures ranging from 80 to 800 K, are presented in Fig. 1B. To explore the potential of the temperature-related optical features of the material studied for temperature sensing applications (luminescence thermometry), we examined several thermometric parameters, such as spectral shift of the emission band, its width, and intensity ratio, whose monotonic evolutions with temperature were studied in detail, confirming good thermometric performance of the material studied, as shown in the Supplementary Text and figs. S7 and S8.

Figure 1C shows the normalized dependence of integrated PL intensity on temperature. As the temperature increases, the PL intensity initially rises to a maximum in the 520 to 540 K range, then declines sharply at higher temperatures. Such a course of the PL thermal quenching curve, in which elevating temperature within a specific range increases PL intensity, is characteristic of materials exhibiting PersL and has been reported for many other phosphors (*25*–*27*). The increase in PL intensity stems from charge-trapping defects in the crystal host and from thermal and optical detrapping of trapped charges, which then recombine radiatively at the luminescence centers, further increasing PL intensity. The role of charge-trapping defects in shaping the PL thermal quenching curve has been thoroughly discussed by Fritz *et al.* (*28*).

To further explore the mechanisms underlying the various optical phenomena of AlN:Mn^2+^, we compared its normalized TL and PL spectra at 80, 300, and 500 K, as shown in Fig. 1D. Across various temperatures, the TL spectra closely match the PL spectra in both spectral position and shape. At 80 K, the PL spectra consist of a series of sharp overlapping lines arising from the zero-phonon and phonon-assisted ^4^T_1_ → ^6^A_1_ transitions in Mn^2+^ ions. With increasing temperature, transitions involving higher-lying phonon states become more prominent, resulting in broader, smoother emission bands in the PL spectra.

**Fig. 2.**
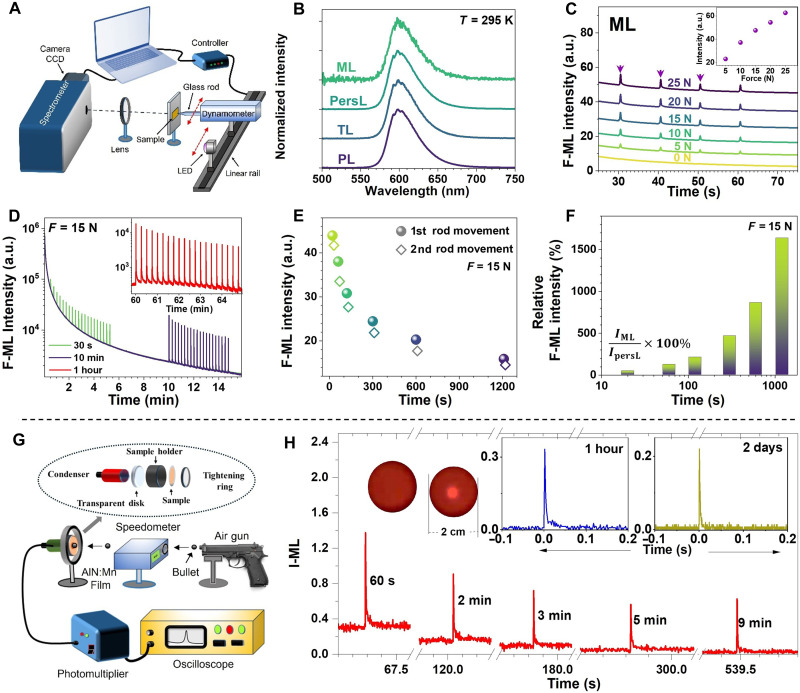
F-ML and I-ML properties of the AlN:Mn^2+^. (**A**) The scheme showing the experimental setup used in the F-ML experiments. (**B**) Comparison of normalized PL, TL, PersL, and ML spectra, measured at room temperature. (**C**) The six curves show the time evolution of the PersL intensity with two ML signals generated for different “waiting times.” Each time, the sample was irradiated with 280 nm light for 2 min. Then, after a set waiting time, the rod, pressed to the sample plate with a force of 15 N, was dragged twice across the plate. The interval between these two movements was 10 s. The inset shows the dependence of the ML signal on the applied force. (**D**) The test of ML sustainability and repeatability, performed for a long series of mechanical stimuli. The F-ML signals were generated with a force of 15 N, and the rod performed 20 movements across the sample every 15 s. The measurements were conducted for waiting time of 30 s, 10 min, and 1 hour. (**E**) Impact of the waiting time on F-ML intensity, induced by the rod’s first and second movement. (**F**) Impact of waiting time on relative F-ML intensity, calculated as percentage emission intensity increase caused by the first movement of the rod. (**G**) Scheme showing the experimental setup used in the I-ML experiments for the AlN:Mn^2+^ sample. (**H**) The curves show the time evolution of the I-ML intensity generated for different waiting times of 60 s, 2 min, 3 min, 5 min, 9 min, 1 hour, and 2 days. The inset photos show the sample before and during impact.

Figure 1E presents the TL glow curves recorded at various heating rates ranging from 0.25 to 2.5 K/s. Before each TL measurement, the sample was preheated to 750 K to empty all charge-trapping states, then cooled to 80 K in the dark. At this temperature, the sample was irradiated with 280 nm light for 2 min and kept in the dark for 1 min before initiating the TL measurement at the specified heating rate. As expected, the higher the heating rate, the greater the absolute intensity of the TL signal, and the signal fades at slightly higher temperatures. Regardless of the heating rate, the TL signal consistently appears as a broad, unstructured band peaking at around 260 K and extending from the lowest measurement temperature up to ~650 K. Such a profile precludes deconvolution into a limited number of discrete trap levels and instead indicates a continuous distribution of trap depths arising from a complex network of defects acting as electron and hole traps. This wide, energetic distribution promotes strong retrapping and charge redistribution even at low temperatures, naturally explaining the pronounced PersL observed in AlN:Mn^2+^ at 10 K and indicating a high density of shallow traps. Detailed TL analyses using the initial rise method and delay experiments further reveal that this continuous trap distribution is accompanied by tunneling-assisted charge detrapping and trap-to-trap redistribution at low temperatures, which substantially contribute to the storage and delayed release of excitation energy (figs. S9 and S10). (*29*, *30*). Despite the presence of numerous trapping states, Mn^2+^ ions act as the sole radiative recombination centers, as confirmed by the good agreement between the ML, TL, and PL emission spectra (see Fig. 2B). At higher Mn^2+^ concentrations, the formation of Mn-Mn pairs and stress-frequency–dependent excitation pathways has been reported to modify the emission characteristics and ML response in Mn-activated phosphors (*31*–*33*); however, such effects are minimized in the low-doping regime used here.

To better understand how UV irradiation time influences the TL response, we measured the integrated TL intensity as a function of irradiation duration (inset of Fig. 1F). After the irradiation stage, the sample was kept in the dark for 60 s, after which the TL signal was measured at a heating rate of 1 K/s. The integrated TL intensity was calculated by integrating the TL glow curves in the 80 to 700 K range. For low UV doses, the integrated TL intensity increases rapidly with exposure time; however, for exposure times longer than 2 min, the increase becomes less steep, tending toward a saturation level. The inset in Fig. 1F shows the normalized TL glow curves measured for different UV irradiation times. At low UV doses, the observed changes in TL glow curve profiles may result from carrier retrapping effects inherent to the material (*34*, *35*). With increasing irradiation time, the TL signal intensity increases, while the overall shape and peak position of the glow curve remain essentially unchanged. This indicates that longer exposure to UV light leads to the filling of more trapping states but does not substantially alter the nature or distribution of the traps themselves. The broad, unstructured nature of the TL glow curve further supports the presence of a wide distribution of trap depths and retrapping processes, as discussed previously. To investigate how the availability of energy stored in trap states changes over time under conditions similar to those used in ML experiments, we also performed TL measurements starting from room temperature. Figure 1G shows the TL glow curves recorded after various waiting times following UV irradiation. The sample was first preheated to 750 K to empty all traps, cooled to 295 K, irradiated with 280 nm light for 2 min, and then kept in the dark for a designated waiting time before TL measurement at a heating rate of 1 K/s. With increasing waiting time, the TL peak gradually shifts toward higher temperatures, while the overall TL intensity decreases. This behavior suggests that, over time, the charges stored in shallow traps are progressively released even at room temperature, leading to their partial depletion before the TL readout. As a result, the remaining stored energy is increasingly associated with deeper traps that require higher thermal energy for detrapping, leading to a shift in the glow peak. The monotonic decrease in TL intensity indicates a continuous loss of stored charge carriers due to spontaneous recombination or slow thermal release from shallow states.

To discuss the mechanism governing the spectroscopic properties of AlN:Mn^2+^ materials in depth, an energy level diagram of the system is presented in Fig. 1H. Such a mechanism is plausibly related to the presence of a continuous distribution of defect-related trap states within the bandgap of the material. The most important defect in the AlN lattice is the O_N_-V_Al_ complex, whose excited state lies close to the conduction band (*36*). Such a configuration can facilitate charge transfer and recombination processes involving Mn^2+^ ions, thereby strongly affecting the emission dynamics and the efficiency of carrier-mediated luminescence. Among the other possible trapping centers, Si-related DX centers (i.e., deep trap states) can also be considered as potential contributors to the observed behavior. Although the Si content in the sample is expected to be very low (as confirmed by the x-ray photoelectron spectroscopy spectrum in fig. S6), as Si was used solely as an oxygen getter and likely vaporized as SiO during synthesis, even trace amounts of Si may lead to the formation of such centers. Their ability to switch between a shallow donor state and a deep DX configuration under external stimuli (light, temperature, or electric field) could thus serve as a plausible microscopic mechanism influencing carrier trapping and luminescence behavior (*37*–*41*).

The trap levels can capture electrons or holes excited by UV or visible light (*42*). Subsequently, the trapped charge carriers can be released into the conduction or valence band, where they migrate to luminescent centers, typically dopant ions, and radiatively recombine, resulting in light emission. If the trap levels of defects are located sufficiently close to luminescent centers, the probability of another release mechanism, quantum tunneling, increases. In this process, charge carriers transition directly from the trap states to the luminescent centers. The essential condition that influences the “typical” luminescence kinetics of Mn^2+^ and gives rise to afterglow luminescence is the presence of trap states that can bind electrons. Electrons excited to the conduction band can be trapped in traps in the AlN lattice. The electrons can be thermally depopulated from the traps, as evidenced by the appearance of a long-lasting, temperature-dependent luminescence. In addition to thermal activation, mechanical stimulation can facilitate trap depopulation by locally deforming the crystal lattice and inducing a piezoelectric effect, thereby promoting the release of trapped charge carriers and altering luminescence behavior.

### Friction- and impact-induced ML

The abundance of trapping states gives the AlN:Mn^2+^ an exceptional ability to accumulate excitation energy, which is then gradually released as intense, PersL, observable already at low temperatures and lasting several hours at room temperature. The high density of charge-trapping states and their energy distribution enable retrapping processes, and the piezoelectricity of the crystal host endows AlN:Mn^2+^ with excellent mechanoluminescent properties. The tested material exhibits immediate, strong, and renewable ML when subjected to mechanical loading. Mn^2+^-doping substantially enhances the ML intensity of the final material compared to the ML of the pure AlN host (producing weak blue ML), plausibly via efficient energy transfer from the host to the activator ions and improved radiative relaxation of the trapped electrons mediated by Mn^2+^. The corresponding results and related discussion are presented in the Supplementary Text and fig. S11.

The experimental details of the following friction-induced ML (F-ML), impact-induced ML (I-ML), and ultrasound-induced ML (US-ML) are presented in the Supplementary Materials.

The AlN:Mn^2+^ powder sample was meticulously and uniformly spread on PP tape, forming a layer approximately 30 μm thick, as mentioned above (see fig. S4). The prepared sample sheet was fixed to the poly(methyl methacrylate) (PMMA) plate and placed in the F-ML setup. We conducted F-ML experiments using a custom-built setup, shown in Fig. 2A, where a glass rod is dragged over the sample surface, which has previously demonstrated excellent performance in the investigation of various other mechanoluminescent materials (*43*–*45*). To identify the intrinsic mechanism of the materials, we compared the normalized spectra of ML, PersL, TL, and PL measured at room temperature, as shown in Fig. 2B. Each spectrum consists of a single band extending from 560 to 700 nm, peaking around 600 nm, with the vibronic structure barely visible near the maximum. The high similarity among the ML, PersL, and TL spectra indicates that the same radiative recombination centers are involved in all three phenomena. Furthermore, the similarity of the ML, TL, and PersL spectra to the PL spectrum demonstrates that Mn^2+^ dopant ions constitute the only recombination centers through which energy stored in the trapping states is radiatively released. At room temperature, the occupancy of shallow trapping states, initially populated through UV irradiation, gradually diminishes over time due to possible nonradiative processes and the strong PersL observed in AlN:Mn^2+^. Both processes decrease the energy stored in the trapping states, thereby reducing the energy available for release under mechanical loads, as observed in ML.

Figure 2C shows the time evolutions of the PersL for the tested sample, superimposed with F-ML (sharp peaks) recorded at different force values (0 to 25 N). The remaining measurement conditions for this experiment are provided in the Supplementary Materials. Arrows indicate the moments when force was applied to the sample. Each mechanical load induced an instantaneous, strong ML signal, observed against a background of a decreasing PersL signal. As shown in the inset of Fig. 2C, to investigate the dependence of F-ML intensity on applied force, a glass rod was pressed against the sample plate with preset forces ranging from 0 to 25 N and dragged across the plate at a constant speed, four times at 10-s intervals. The increased applied force led to a proportionally higher ML intensity. The range and shape of the ML spectrum remained independent of mechanical loading. The F-ML intensity was calculated as the excess in the measured emission intensity over the PersL signal.

Figure 2D shows the F-ML induced by a long series of mechanical stimuli. Before each experiment, the sample plate was irradiated with 280-nm light for 2 min. The F-ML signals were generated with a force of 15 N, and the rod performed 20 movements across the sample every 15 s, starting 30 s, 10 min, and 1 hour after the irradiation step was completed. The rod drew on the same part of the sample plate, generating a robust ML signal with each successive movement of the long series. Figure 2E summarizes the impact of “waiting time” on the F-ML intensity induced by the rod’s first and second movements. Figure 2F illustrates the effect of “waiting time” on the relative F-ML intensity, calculated as the percentage increase in emission intensity caused by the rod’s first movement. Even as the PersL signal diminishes, the tested sample maintains a strong and stable ML response. This indicates that in the case of AlN:Mn^2+^, the ML process involves charge carriers from shallower traps, which contribute to the PersL signal observed at room temperature, as well as charges trapped in deeper states. Most of the released charges lead to immediate emission, as little afterglow is generated after friction-induced release. A more detailed discussion on the ML-active traps refilling (after the emission event) is provided in the Supplementary Materials. Furthermore, the hopping of charges from deeper traps to shallower ones, and subsequently to Mn^2+^ ions, where they radiatively recombine, could be facilitated by a reduction in the energy barriers for electrons and holes to escape from traps due to mechanical loading of the sample. This effect may be further enhanced by the piezoelectric fields generated within the wurtzite AlN matrix under stress, which locally modulate the potential landscape and promote charge release and migration. Such a mechanism could explain the renewable character of the ML observed in the material. In addition, we performed a piezoelectric performance test, allowing determination of the piezoelectric coefficient (*d*_33_) for the pure AlN single crystal, yielding a *d*_33_ value of ≈4.6 pC/N, which agrees well with literature data for different AlN materials reported elsewhere (*46*–*48*). Note, that the polycrystalline AlN:Mn^2+^ powder material, studied in this work, consists of randomly oriented grains, in which any anisotropic piezoelectric effect is averaged out. The experimental details and the related discussion are given in the Supplementary Materials.

To check the minimum detectable stress/pressure that could generate a measurable ML signal, we placed a sample plate in the F-ML measurement setup and stored in a darkness for 1 week. After this period, a series of measurements was performed. We found that even the smallest force reliably measurable in our setup, namely, 0.2 N, generated a clear and readily detectable ML signal (see fig. S12). The estimated contact area between the glass rod and the sample was 0.25 mm^2^, corresponding to a pressure of approximately 8 bar (0.8 MPa). Although, because of the technical reasons, we could not determine the absolute sensitivity limit, i.e., the minimum load required to induce ML, our results indicate that in this case the limit of detection is lower than 8 bar.

I-ML serves as a vital probe of transient mechanical events, providing valuable insights into the real-time response of materials to sudden stress (*49*). The I-ML experimental setup is shown in Fig. 2G. It is demonstrated in Fig. 2H that AlN:Mn^2+^ exhibits intense and persistent ML, even long after UV irradiation. ML signals were generated by identical spherical projectiles striking the sample. The projectiles were fired from a gun after the specified time following the one-time UV irradiation exposure, as listed in Fig. 2H. Despite all projectiles notable the same spot on the sample, each subsequent strike induced an instantaneous, short-lasting, but marked increase in the intensity of the emitted light over the background of the fading PersL signal. It is worth noting that the I-ML signal remained measurable even 2 days after UV irradiation. Before the projectile struck the sample, the entire surface emitted a homogeneous orange-red PersL, indicating a uniform distribution of AlN:Mn^2+^ powder in the film. However, the bright, intense I-ML response was confined to the area of the sample film struck by the projectile, demonstrating the material’s potential application for precise, remote, and real-time impact detection.

It is worth noting, that in principle, uniform stress during compression or stretching primarily induce volume-driven polarization (piezoelectrification), whereas friction mainly introduces surface-driven charge transfer (triboelectrification), often accompanied by highly nonuniform, transient electric fields. In the case of impact, both piezoelectric and triboelectric mechanisms may contribute to ML, as the process involves not only bulk lattice deformation but also rapid contact-separation events and possible surface charge transfer. Triboelectric processes involve real charge exchange and accumulation, while piezoelectricity involves the generation of bound polarization charges without net charge transfer across interfaces. However, in real scenarios, as in our case, during F-ML experiments there is also a substantial contribution from the piezoelectric effect, whereby the movable rod not only scratches the surface of the ML-active material but also induces stress through pressing and stretching as it moves. Hence, in such cases, the synergistic effects of both mechanisms, which are inherently coupled and difficult to analyze separately, are expected to occur simultaneously.

### Ultrasound-induced ML

**Fig. 3.**
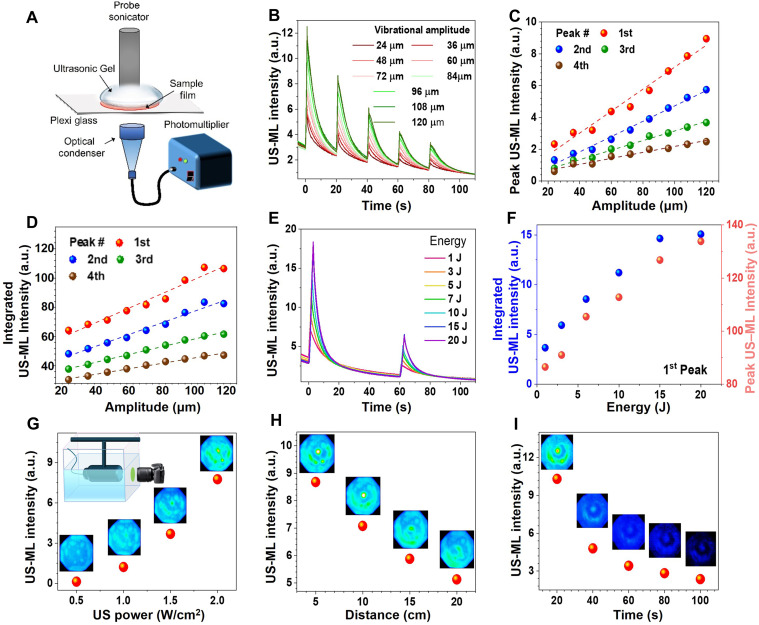
Sound-induced ML phenomena in the AlN:Mn^2+^ material. (**A**) The schematic illustration of the US-ML setup, operating at 20 kHz. (**B**) Intensity of US-ML versus time at 1/19 on/off ratio and various vibration amplitudes from 24 to 120 μm with a step size of 12 μm. US-MLs are measured at 20 kHz. (**C**) Peak intensity of US-ML for first, second, third, and fourth cycle versus amplitude of probe vibration. (**D**) Integrated US-ML intensities for first, second, third, and fourth cycle versus amplitude of probe vibration. (**E**) Time dependence of US-ML intensity, measured at different energies of US exposure from 1 to 20 J and fixed 60-μm vibrational amplitude. (**F**) Peak US-ML and integrated US-ML as a function of US energy, calculated for the first cycle. (**G**) Dependence of US-ML on the applied ultrasound power. The distance between the ultrasound transducer and the sample sheet was 3 cm. US-ML was measured at 3.3 MHz. The water temperature used as the ultrasound transmitting medium was 20°C. The inset shows a schematic illustration of the US-ML setup, using 3.3 MHz. (**H**) Dependence of US-ML on the distance between the ultrasound transducer and the sample sheet, measured at 2 W/cm^2^ ultrasound power. (**I**) The dependence of US-ML on time was measured at 2 W/cm^2^ ultrasound power and a 3 cm distance between the transducer and the sample sheet.

US-ML is a powerful technique for probing material responses to high-frequency mechanical stimulation, enabling noncontact, localized activation of luminescent behavior. As discussed above, the high sensitivity and reproducibility of F-ML and I-ML make AlN:Mn^2+^ a promising material for a wide range of applications. To comprehensively assess its ML behavior, we further explored its capability in US-ML. Two experimental setups and multiple approaches were used to test US-ML at 20 kHz and 3.3 MHz. The experimental setup for US-ML, operated at 20 kHz, is shown in Fig. 3A, and the US-ML results at 20 kHz are shown in Fig. 3 (B to F) and fig. S13. Figure 3B shows the time dependence of the US-ML intensity measured at various probe sonicator vibrational amplitudes, ranging from 24 to 120 μm, with a step size of 12 μm. The US-ML was generated in five cycles, each lasting 20 s, with a sonication-on/off time ratio of 1/19 s. Figure 3C shows the dependence of US-ML peak intensities on the vibrational amplitude for the first four of five applied cycles. Figure 3D shows the integrated US-ML intensity, calculated by integrating the US-ML intensity over time. In both cases, the US-ML intensities were calculated as the US-induced signal excess relative to the PersL signal without ultrasound stimulation. The peak intensity and the integrated US-ML intensity are linearly dependent on the vibrational amplitude, with the slope decreasing for each subsequent cycle. Despite the relatively short exposure time of the sample to the US beam, the increase in vibration amplitude could also cause a slight rise in the temperature of the sonicator probe tip, and consequently, in the ultrasound-transmitting gel and the sample sheet. The apparent consequence of the temperature increase in AlN:Mn^2+^ is a higher probability of charge carriers escaping from trap states. Nevertheless, the higher vibration amplitude also led to a greater force applied to the sample, resulting in greater deformation and a more pronounced ML response.

In the second test, the US-ML was measured as a function of the applied US energy, ranging from 1 to 20 J. During these measurements, the probe tip vibrated with an amplitude of 60 μm, and the sample sheet was exposed to ultrasound for automatically adjusted exposure times to achieve the preset US energy values. Figure 3E shows the time evolution of US-ML intensity, measured at various US energies over two on/off cycles, each lasting 60 s. Figure 3F shows the impact of US energy on US-ML peak intensity and integrated US-ML intensity, calculated for the first cycle, based on the experimental curves presented in Fig. 3E.

In the third approach performed at 20 kHz, we tested the impact of sonication time on US-ML intensity, as presented in fig. S13. The US-ML was generated in five cycles, each lasting 20 s, at a constant vibrational amplitude of 24 μm, with varying sonication on/off time ratios. Figure S13A shows the time evolution of US-ML measured for different US on/off time ratios. The US-ML intensity increased with the duration of US stimulation; however, the longer the US on-time, the more pronounced the saturation effect in the US-ML signal became, as evidenced by a deviation from the linear dependence of the US-ML signal rise on the exposure time to US within a given cycle. It was observed for all cycles in the series, although the US-ML intensity decreased over successive cycles for each set of on/off ratios. Figure S13B shows the peak US-ML intensity as a function of the on-duration of US in each cycle. The peak US-ML intensity was calculated as the ultrasound-induced increase in the sample’s light intensity relative to the decaying PersL signal measured before ultrasound stimulation. Figure S13C shows the integrated US-ML intensity as a function of the on-duration of each US pulse, calculated by integrating the US-ML signals over time.

Figure 3 (G to I) summarizes the results of the US-ML experiments conducted at 3.3 MHz, a frequency commonly used in medical and physiotherapeutic devices. The graphs display the measured US-ML intensities, represented by symbols. The insets above the symbols also show the corresponding camera frames used to determine the US-ML intensities. These images reveal the impact of the altered parameter on the spatial distribution of the US-ML signal across the sample sheet. The inset in Fig. 3G shows the setup used for US-ML at 3.3 MHz. Figure 3G presents the results of the first approach, which examined the relationship between the power of the applied US waves (ranging from 0.5 to 2 W/cm^2^) and the resulting US-ML intensity. The distance between the ultrasound transducer and the sample sheet was 3 cm. For each preset US power level, the sample was first irradiated with UV light for 1 min, then kept in the dark for 20 s, and thereafter exposed to the US beam for 10 s. The results show a nonlinear increase in US-ML intensity with rising US wave power. The next test measured the dependence of US-ML intensity on the distance between the US transducer and the sample sheet. After each distance adjustment, the sample was irradiated with UV light for 1 min, kept in the dark for 20 s, and then exposed to US waves for 10 s. As shown in Fig. 3H, the US-ML intensity decreases with increasing distance from 5 to 20 cm. A change in the distribution of the US-ML signal intensity across the sample sheet also accompanies this (*50*). As shown in Fig. 3I, to investigate the reproducibility of the US-ML, the sample sheet was first UV-irradiated for 1 min, then kept in the dark for 20 s, and subsequently subjected to a series of five 10-s exposures to US, with 10-s time gaps between them. The distance between the ultrasound transducer and the sample was 3 cm, and the US power was 2 W/cm^2^.

Notably, such excellent US-ML properties of AlN:Mn^2+^ enable its potential application in noninvasive biomedical imaging, particularly for deep-tissue visualization, where optical access is limited. This is because, for the material studied we demonstrate a detectable ML signal at pressures on the order of ~0.8 MPa, located within acoustic pressures encountered in biological tissues during ultrasound exposure, with the actual sensitivity limit expected to be lower. Specifically, this pressure range overlaps with the upper range of acoustic pressures encountered in ultrasound-based techniques, where peak pressure amplitudes can reach sub-MPa to MPa levels, depending on frequency and intensity.

Its sensitivity to high-frequency mechanical waves also makes it suitable for integration into US structural health monitoring systems, enabling real-time detection of stress or damage in critical components. In addition, US-ML from AlN:Mn^2+^ can be used to remotely activate light-emitting devices or sensors in enclosed or hard-to-reach environments, such as sealed containers or implanted medical devices. Given its unique combination of remote activation, high sensitivity, and environmental adaptability, the US-ML functionality of AlN:Mn^2+^ holds great promise for future development in innovative diagnostics, wireless sensing, and advanced optoelectronic systems.

**Fig. 4.**
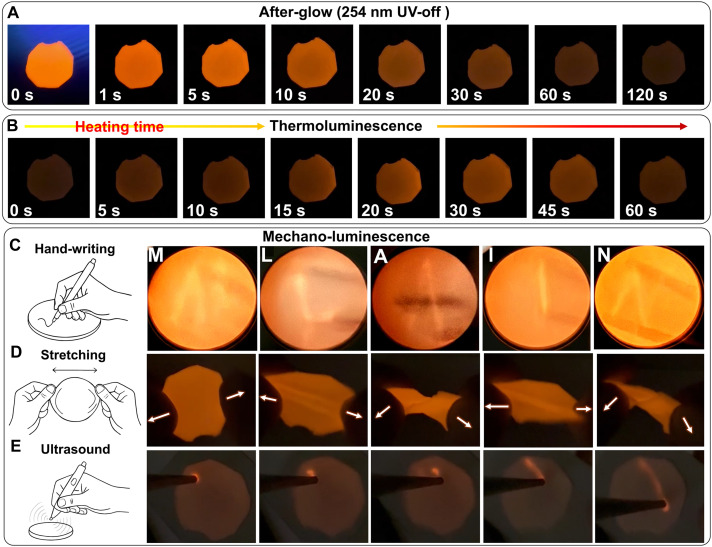
Visualization and application potential of the PL-, TL-, and ML-active AlN:Mn^2+^-modified PDMS film. (**A**) Afterglow emission, (**B**) TL, and (**C** to **E**) ML-based applications of the AlN:Mn^2+^-modified PDMS film. Bright orange ML effects in [(C) to (E)] were induced with different stimuli, i.e., friction (C), stretching (D), and ultrasounds (E), using a pen-like metal rod, hands, and FUS device, respectively.

To showcase the application potential of the developed material, we embedded the AlN:Mn^2+^ powder (10 weight %) into the polymer resin, polydimethylsiloxane (PDMS), and fabricated the AlN:Mn^2+^-modified thin film (≈0.8 mm) of PDMS in a form of rounded-shape disc (≈3 cm in diameter), which combines luminescence features of the inorganic modifier and elasticity of the organic polymer. Figure 4A shows bright orange emission of the fabricated composite film upon 254-nm UV lamp irradiation (0 s), and its long-lasting afterglow emission (UV-off), visible even a few minutes after irradiation. The related TL emission of the deep-trapped electrons induced by external heating (≈500 K) is shown in Fig. 4B. The most appealing ML phenomena of the AlN:Mn^2+^-modified polymer film are presented in Fig. 4 (C to E), which depict bright orange ML emissions induced by different stimuli, namely friction (C), stretching (D), and ultrasound (E). Thanks to the ML effect, the handwritten letters “M,” “L,” “A,” “I,” and “N” (created with a pen-like metal rod) are visible as brighter areas on the pre-irradiated film (Fig. 4C). The stretching and bending patterns (Fig. 4D) can be easily recognized as brighter areas/strips in the hand-stretched film, allowing visualization of tensile and shearing stresses within, where the effects of the interface between the PDMS and the phosphor particles can play a role (*51*). Last, the FUS-induced ML is clearly visible (Fig. 4E) as long-lasting, shining patterns, revealing the intriguing possibility of sound visualization. The discussed effects can also be observed in the recorded videos (movies S1 to S6), provided in the Supplementary Materials. To help visualize the material’s macroscopic homogeneity and microscopic structure, photographs of the composite film under white-light illumination and the corresponding optical micrograph are provided in fig. S14, confirming the uniform distribution of the luminescent particles within the polymer matrix. Overall, the presented application potential confirms the outstanding ML performance and utility of the developed material for advanced anticounterfeiting, handwriting style recognition, optical coding, night-vision safety signs, and force and sound visualizations.

## DISCUSSION

In summary, we have demonstrated that Mn^2+^-doped aluminum nitride (AlN:Mn^2+^) serves as a multifunctional luminescent material, combining PersL/PL, TL, and force- and sound-induced ML within a single material, opening new horizons in modern optoelectronics and advanced energy transformation. Its ability to respond to a wide range of external stimuli, including temperature, mechanical force (friction and impact), and high-frequency acoustic waves, enables AlN:Mn^2+^ to function effectively as both a mechanoluminescent force sensor and a high-sensitivity, PL-based temperature sensor. The material supports advanced thermometry techniques through band-ratio, centroid, and bandwidth analysis. The convergence of these optical properties in a single, robust, and chemically stable material highlights the considerable potential of AlN:Mn^2+^ for future applications in smart sensing, biomedical diagnostics, structural health monitoring, and adaptive optoelectronic systems. This work paves the way for the design of next-generation multifunctional luminescent platforms capable of operating in diverse and demanding environments, allowing the conversion of different types of energy, including mechanical and acoustic waves, into electromagnetic waves.

## MATERIALS AND METHODS

### Preparation procedure of AlN:Mn powder

The synthesis procedure of the tested sample, i.e., AlN:Mn^2+^ 0.06 mol %, was described in detail in (*21*), and the material this work used comes from the same batch. In brief, the sample was synthesized as follows: Commercial aluminum nitride powder was ground with Mn and Si dopants in an agate mortar, then placed in a boron nitride crucible and sintered under 10 to 100 atm of nitrogen pressure at 1900°C for 2 to 4 hours. In the next step, the powder was ground again and annealed in a flowing nitrogen atmosphere at 1650°C. Si is not incorporated into the powder but serves as an oxygen getter, vaporizing as SiO during the synthesis. Note, that for the further experiments the as-synthesized material was handled in the as-received form, without any further particles sieving or subjecting to any other post-treatment.

### Preparation procedure of AlN:Mn in plate/sheet

For F-ML and I-ML studies, an even layer of AlN:Mn powder was affixed to a PMMA plate using specially selected UV-transparent PP adhesive tape. A PDMS sheet with a thickness of 0.8 mm and a diameter of 3 cm, containing uniformly distributed AlN:Mn powder, was prepared for the US-ML measurements. The sheet was fabricated by thoroughly mixing 0.5 g of PDMS base with 0.05 g of AlN:Mn powder, followed by the addition of 0.06 g of curing agent. The resulting mixture was poured into a mold and cured at 60°C for 24 hours.

### Characterizations

#### Structural characterizations

The powder XRD measurement was made using a BRUKER D2PHASER using Cu radiation operating at 30 kV and 10 mA. The XRD pattern was collected for 2θ values ranging from 10° to 80°, with a scan step of 0.02° and a counting time of 0.4 s per step. The phase analysis was conducted using DIFFRAC.EVA software from BRUKER. The morphology of the sample was tested using a TM-1000 SEM from Hitachi. The Raman spectra were recorded using a confocal micro-Raman system (Renishaw InVia), equipped with a 532-nm power-adjustable laser.

#### Optical characterizations

For the TL and temperature-dependent PL measurements, the THMS600 Linkam stage temperature controller, along with the LNP95 liquid nitrogen cooling pump system, was used. An LED light at a wavelength of 280 nm served as the excitation source. The TL and PL signals were collected using the Andor SR-750-D1 spectrometer, equipped with a charge-coupled device (CCD) camera (DU420A-OE). To investigate the trap depth distribution and tunneling effects, additional TL experiments were performed using a Linkam FTIRSP600 temperature stage equipped with an LNP96 module to cool down to 100 K. Excitation was performed by means of a UV-C (3 W) lamp. The phosphor’s emission was led via an optical fiber to an electron-multiplying CCD camera (Princeton Instruments ProEM 16002) connected to a spectrograph (Princeton Instruments Acton SP2358).

#### Friction-induced mechanoluminescence

The F-ML was measured using a custom-built setup controlled by dedicated software in LabVIEW. The setup included a 280-nm emitting light diode as the excitation source and a force gauge combined with a glass rod, mounted on a linear rail. The ML signal was acquired with a spectrometer (Shamrock SR-500i) equipped with a CCD camera (iDus420). To induce F-ML, the glass rod was pressed against the sample plate with a preset force and dragged across the plate at a specified speed and frequency. All F-ML measurements were conducted at temperature 293 K.

#### Impact-induced mechanoluminescence

The I-ML measurements were conducted using a setup detailed in (*49*). This setup includes an airsoft electric gun (model CM.122), a speedometer, a cylinder with a target plate, and a detection system comprising a photomultiplier tube (H10721P-04) and an oscilloscope (WaveSurfer 432). Before the I-ML experiment, the sample, fixed to the PMMA target plate, was exposed to 270-nm light from a diode for 1 min. ML was then induced by shooting a spherical projectile with a 6-mm caliber and a mass of 0.25 g at a velocity of 53 ± 3 m/s from a distance of 20 cm.

#### US-ML measurements at 20 kHz frequency

The US-ML measurements at 20 kHz frequency were performed using an US probe sonicator (model: CV334, vibracell, SONICS) and a photomultiplier (model: H10720P-04) connected with a multimeter (Keithley 2000). The US probe, with a tip diameter of 13 mm, was mounted perpendicularly to the sample sheet at a distance of 1 mm. The device allows setting the amplitude of the probe tip vibrations between 24 and 120 μm, and the wattage required to maintain the preset amplitude is adjusted automatically. The space between the probe tip and the sample sheet was filled with a commercial medical ultrasound transmitting gel. Before the US-ML experiment, the sample was irradiated with 270-nm light from a diodes array for 1 min. Then, the sample was kept in the dark for 1 min before exposure to US.

#### US-ML measurements at 3.3 MHz frequency

The US-ML measurements at 3.3 MHz frequency were executed using a physiotherapeutic Gymna 200 piston transducer emitting 3.3 MHz frequency US waves with a power ranging from 0.1 to 2.0 W/cm^2^. The transducer was mounted on a movable handle inside a 5-liter water-filled tank and positioned perpendicularly to the sample sheet fixed to the tank wall. The US-ML was measured using a Ximea MC031MG-SY-UB camera equipped with a Sony CMOS sensor chip (3.1 MPix) and an Edmund Optics lens with a focal length of 3.5 mm (TechSpec C Series). The frames were collected at a rate of 5 fps (exposure time of 200 ms). In this series of experiments, the sample sheet was irradiated with 270-nm light from a diode, and then after 20 s in the dark, the sample was exposed to US beam for 10 s. The US-ML intensity was derived using the public domain software ImageJ as the average of the signals recorded by the camera sensor’s pixels. The US-ML intensity was calculated as the difference between the light intensity measured immediately before and during the sample’s exposure to US.

## Supplementary Materials

### The PDF file includes:

Supplementary Text Figs. S1 to S14 Table S1 Legends for movies S1 to S6 References
