# Supplementary material for: Turning sound and force into light with AlN:Mn2+ mechanoluminescence
Source: Sci Adv. 2026 Jun 26;12(26):eaed5469. doi: 10.1126/sciadv.aed5469 (PMC13308619; doi:10.1126/sciadv.aed5469)
Supplement: Supplementary file 1 — Supplementary Text Figs. S1 to S14 Table S1 Legends for movies S1 to S6 References [file sciadv.aed5469_sm.pdf]

Science Advances
AAAS

Supplementary Materials for

Turning sound and force into light with AlN:Mn^{2+} mechanoluminescence

Justyna Barzowska *et al.*

Corresponding author: Teng Zheng, zhengteng@hzcu.edu.cn; Marcin Runowski, runowski@amu.edu.pl; Sebastian Mahlik, sebastian.mahlik@ug.edu.pl

Sci. Adv. **12**, eaed5469 (2026)
DOI: 10.1126/sciadv.aed5469

The PDF file includes:

Supplementary Text
Figs. S1 to S14
Table S1
Legends for movies S1 to S6
References

Other Supplementary Material for this manuscript includes the following:

Movies S1 to S6

Supplementary Text S1: Concentration and valence state of Mn^{2+} dopant ions

In the present study, the Mn^{2+} concentration was deliberately chosen to maximize the luminescence intensity, while simultaneously avoiding concentration quenching and Mn–Mn interactions. Consequently, the observed mechanoluminescence and persistent luminescence are dominated by isolated Mn^{2+} centers. This interpretation is consistent with the absence of additional emission features and with the close correspondence between PL, TL and ML spectra. Note, that the sample with very low concentration of Mn^{2+} of 0.06 mol.% exhibits the best luminescence performance (within the initially synthesized series of Mn^{2+}-doped samples, spanning from 0.01 to 0.55 mol.% of Mn^{2+}), i.e. the strongest luminescence intensity, resulting from the highest quantum yield (QY = 0.86%), as reported in our previous work (*21*).

Note, that because the sample studied here, i.e. AlN:Mn^{2+} 0.06 mol.%, has been taken from the same batch of Mn^{2+}-doped aluminum nitrides (ref. (*21*)), where Mn^{2+} concentration spanned from 0.01 to 0.55 mol.%, in this work we performed only the basic measurements confirming long-term chemical stability of the material (after ca. 10 years since its first fabrication), including XRD, Raman spectra, EDX, XPS and SEM. The exact concentration of Mn^{2+} in the synthesized material was determine with Glow Discharge Mass Spectrometry (GDMS) method, and the valence state of Mn^{2+} was undoubtedly confirmed by the X-ray Absorption Near Edge Structure (XANES) method in our previous work (*21*). Note, that due to the very low concentration of manganese in the synthesized material, its presence could not be detected neither by the EDX nor by the XPS methods, which are less sensitive, compared to the GDMS and XANES. That is why, the presence of manganese ions is not manifested in the recorded EDX and XPS spectra in Figs. S5 and S6, respectively. It is worth noting, that in the XPS spectrum of the synthesized AlN:Mn^{2+} material (Fig. S6), the postulated trace amounts of Silicon (Si), related to the formation of deep trap states (DX centers) can be observed as two very low intense peaks. Note, the peaks assigned to calcium (Ca) plausibly originate from the dust adsorbed on the particles surface, as frequently found in XPS spectra of other calcium-free compounds.

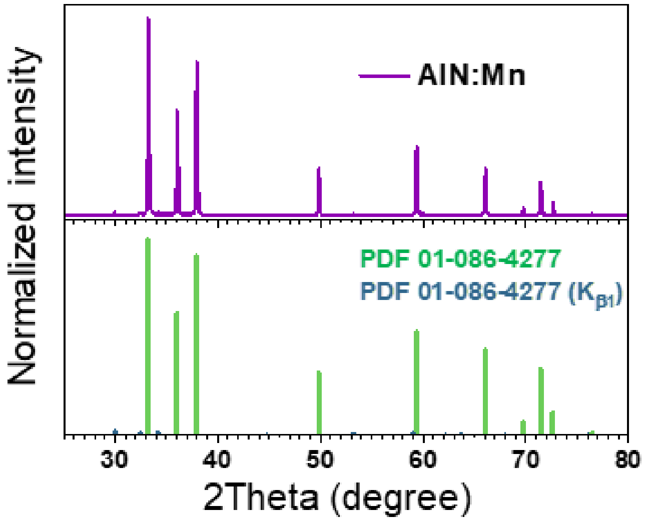

Fig. S1. Powder XRD pattern of the investigated AlN:Mn^{2+} material and wurtzite AlN standard (PDF 01-086-4277).

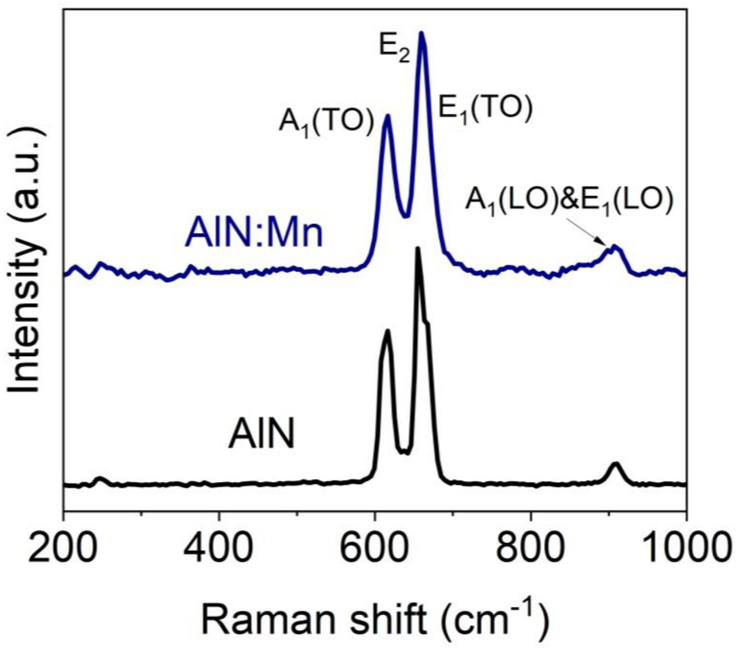

Fig. S2. Raman spectra for the – AlN and AlN:Mn^{2+} material.

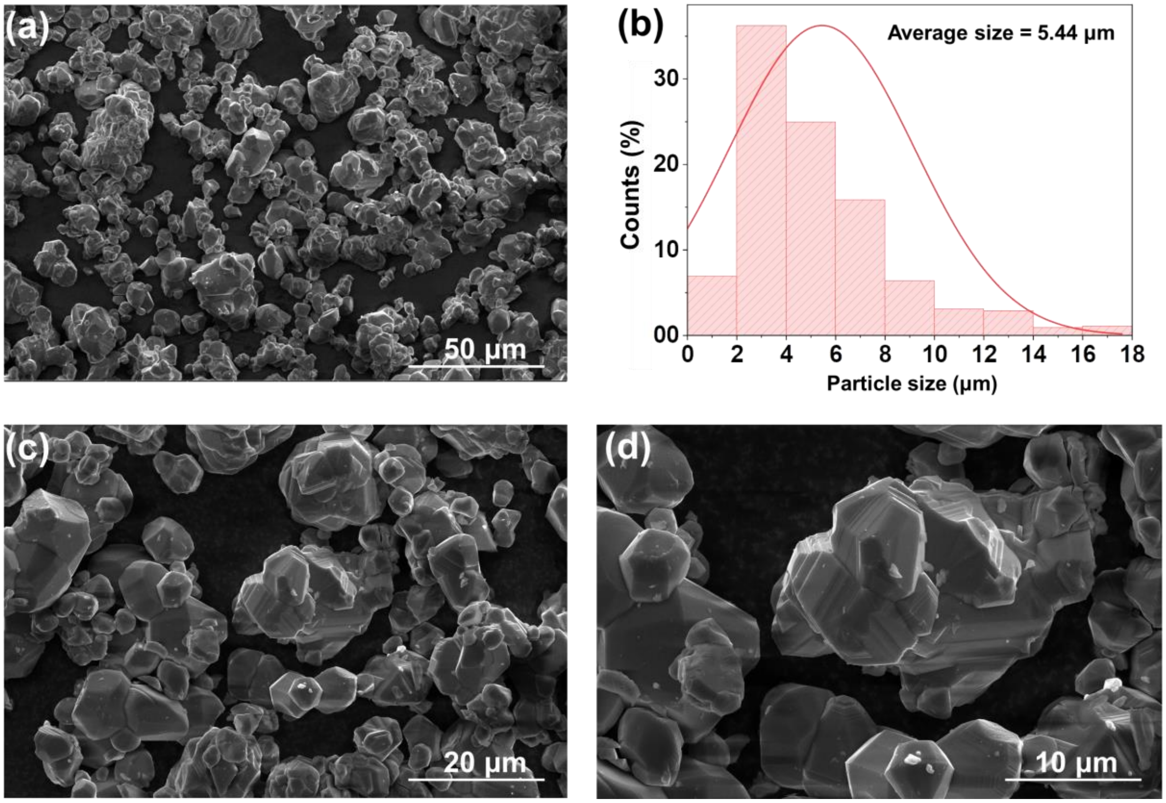

Fig. S3. (a, c, d) SEM images at different magnifications and (b) the corresponding size distribution histogram for the polycrystalline AlN:Mn^{2+} powder.

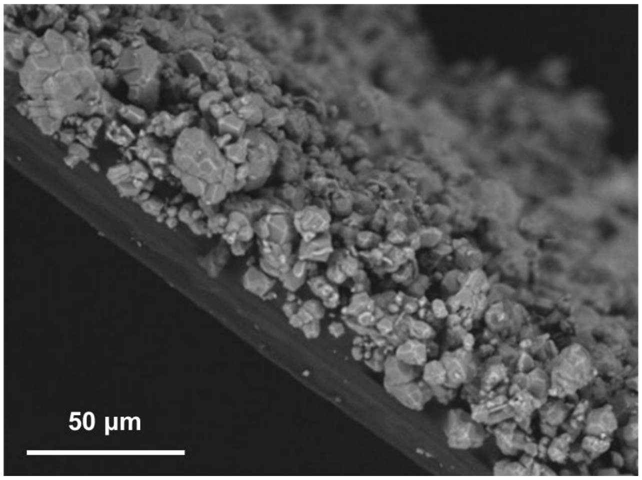

Fig. S4. SEM image of the cross-sectional view of the sample sheet prepared for the F-ML experiment

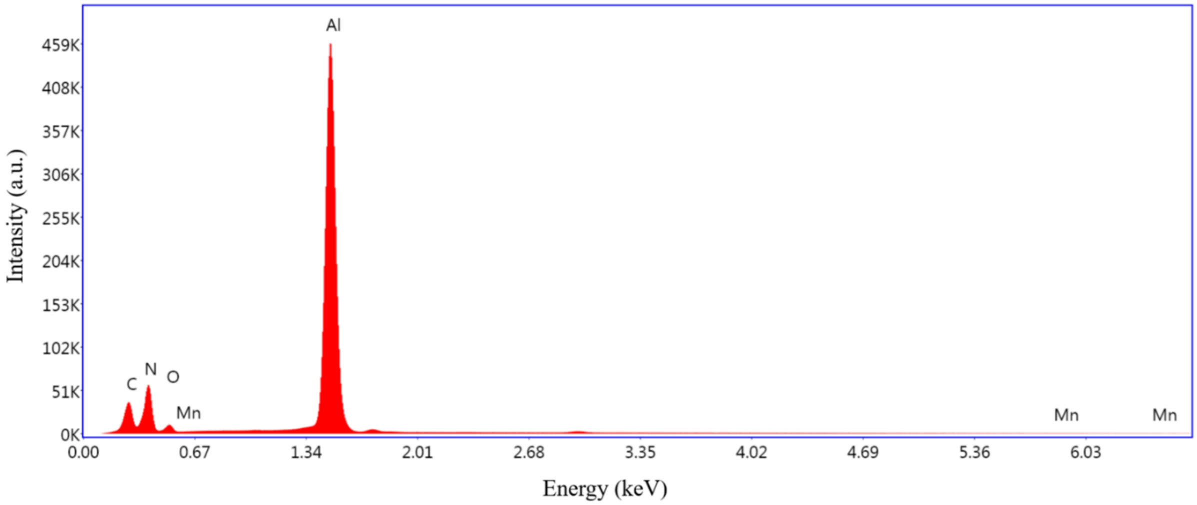

Fig. S5. EDX spectrum of the AlN:Mn^{2+} powder.

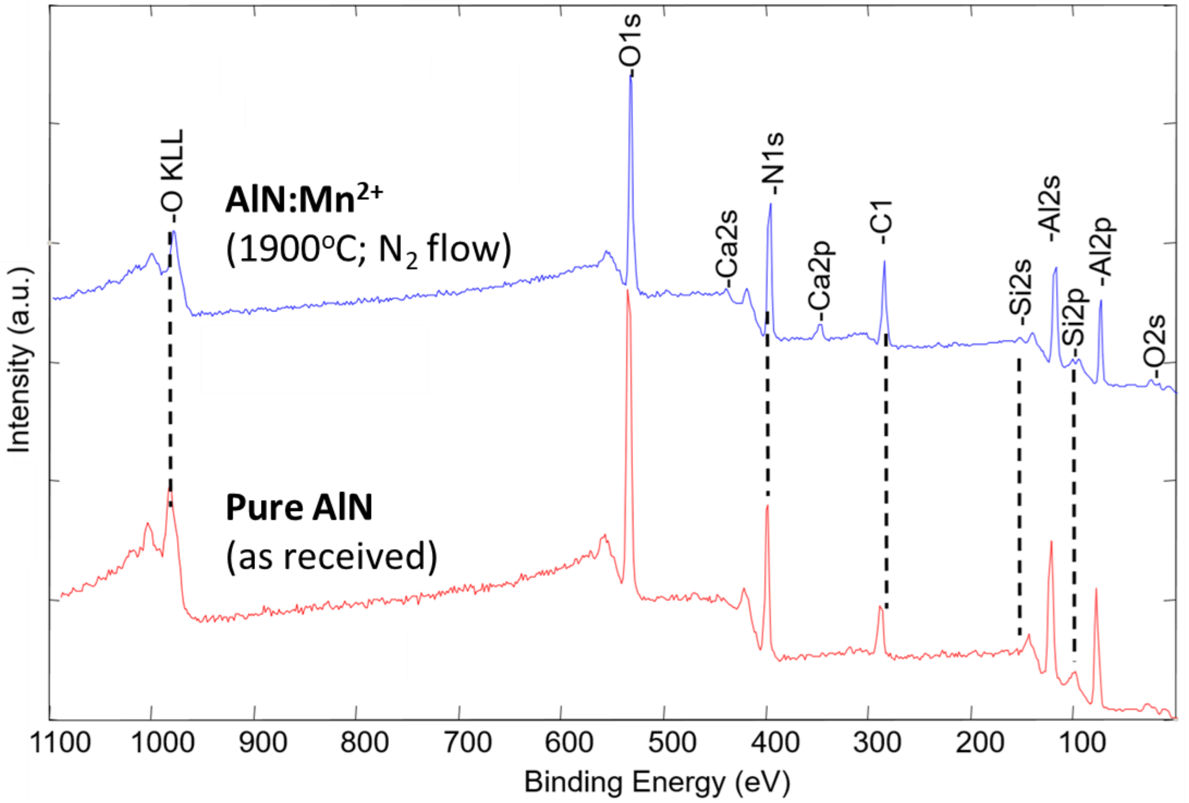

Fig. S6. XPS spectrum of the AlN:Mn^{2+} powder compared with the purchased pure AlN used for the synthesis.

Supplementary Text S2: Luminescence thermometry – temperature sensing properties

To explore the potential of the material for temperature sensing applications, we examined its spectroscopic properties at various temperatures. The normalized PL emission spectra measured at 80 – 460 K are presented in Fig. S9a. As mentioned above, the PL spectra comprise a series of sharp, overlapping lines originating from the zero-phonon transitions, along with a broad band attributed to phonon-assisted $^4T_1 \rightarrow {}^6A_1$ transitions of Mn^{2+} ions. At low temperatures, these sharp peaks are clearly observed near the maximum of the emission band, which can be attributed to vibronic components. In the temperature range of 80 to 460 K, the vibronic features remain prominent, and each component exhibits high sensitivity to temperature variations. Thus, the peaks at 591.5 nm, 597.6 nm, and 603.2 nm are labeled as P_1, P_2, and P_3, respectively. The intensities of these emission peaks were determined by integrating the emission spectra over their respective wavelength ranges. It is clear in Fig. S9b-d that the band ratio of the three vibronic peaks significantly change with temperature. It is worth noting that, to date, the use of vibronic components for temperature sensing has been reported in only one other case (*52*). As shown, the band ratio of P_1/P_3, P_1/P_2 and P_3/P_2 in the temperature range of 80 to 460 K can be well fitted to an exponential function:

$$Band\ ratio = A_1 exp\left(-\frac{T}{t_1}\right) + A_2 exp\left(-\frac{T}{t_2}\right) + y_0 \qquad \text{(S1)}$$

Here, *T* denotes the temperature, and the *band ratio* refers to the ratio of the integrated intensities of the corresponding vibronic peaks. The corresponding fitting parameters, including A_1, t_1, A_2, t_2, y_0, for each band ratio can be found in Table S1 in SI. The most reliable Fig. of merit for sensing based on band intensity ratios (as well as emission lifetimes) is the relative sensitivity (S_r), typically expressed as a percentage. It quantifies the change in the measured parameter per 1 K of absolute temperature (*53*–*56*). The temperature-dependent values of S_r were determined using the following equation:

$$S_r = \frac{1}{band\ ratio}\left|\frac{d(band\ ratio)}{dT}\right| \times 100\ \% \qquad \text{(S2)}$$

As shown in Fig. S9e, it is evident that all S_r values exhibit a decreasing trend with increasing temperature. The maximal S_r = 0.113% K^{-1} (at 80 K), 0.205% K^{-1} (at 80 K) and 0.110% K^{-1} (at 80 K) are achieved for the band ratio of P_1/P_3, P_1/P_2 and P_3/P_2, respectively.

Moreover, when the temperature rises to 480 K or higher, the vibronic components are no longer observable, as shown in Fig. S9f. To assess the potential of the studied material as an optical thermometer, its thermometric performance was first evaluated based on the bandwidth (FWHM) and spectral shift (band centroid) of the phonon-assisted $^4T_1 \rightarrow {}^6A_1$ transitions of Mn^{2+}. Notably, as the temperature increases from 480 to 800 K, the emission band shifts toward shorter wavelengths and becomes progressively broader. Specifically, as the temperature increases from 480 to 800 K, the band centroid shifts from 610.8 to 605.0 nm, while the FWHM increases markedly from 49.6 to 66.8 nm (see Fig. S9g, h). The band centroid and FWHM of the $^4T_1 \rightarrow {}^6A_1$ emission band of Mn^{2+} in the 480–800 K temperature range can be well fitted using the exponential function (1). The sensitivities for band-shift (dλ/dT) and bandwidth (dFWHM/dT) were also calculated using the equation:

$$S_a = \left|\frac{d(centroid)}{dT}\right| \ or \ S_a = \left|\frac{d(FWHM)}{dT}\right| \quad \text{(S3)}$$

As shown in Fig. S9i, the S_a values for both the centroid and FWHM exhibit an increasing trend with rising temperature. The maximum values, S_a =0.027 nm/K for the centroid and S_a =0.074 nm/K for the FWHM, are observed at 800 K. In comparison with other PL thermometers that rely on band shift or bandwidth, i.e., $LaF_3:Nd^{3+}$ (dλ/dT= ~0.0068 nm/K), $Na_4Mg(WO_4)_3:Mn^{4+}$ (dλ/dT= 0.127 nm/K) (*57*), $YAG:Ce^{3+}$(dλ/dT= ~0.047 nm/K) (*58*), $SrB_4O_7:Tm^{2+}$ (dFWHM/dT=0.092 nm/K) (*52*), $YAlO_3$: Nd^{3+}(dFWHM/dT=~0.039 nm/K) (*59*), and Gd_2ZnTiO_6: Mn^{4+} (dFWHM/dT=~0.090 nm/K) (*60*), the $AlN:Mn^{2+}$ based sensor clearly demonstrates notably high absolute sensitivity.

Finally, in order to examine in detail the performance of the proposed temperature sensor, we determined the temperature sensing resolution – δT (uncertainty of temperature readouts) for all thermometric parameters used, i.e. for three different band intensity ratios, emission band centroid and FWHM, and plotted the calculated values as a function of temperature in Fig. S10. The δT values were calculated based on the following equation (Eq. S4):

$$\delta T = \frac{1}{S_r}\frac{\delta MP}{MP} \quad \text{(S4)}$$

where δMP is the uncertainty of the determined measured parameter - MP (i.e. band ratio, centroid or FWHM) value. The use of thermometric parameters based on intensity ratio, operating from the cryogenic T-range, i.e. from 80 K up to 480 K, results in good sensing resolution in the cryogenic range (δT < 1 K), but it significantly deteriorates above room temperature to approximately 2-5 K (depending on the particular band ratio used), and finally reaches around 10 K (or even more for the P_1/P_3 ratio) at the maximum operating temperature, making the potential thermometric readout highly inaccurate. That is why, we recommend to use the emission band centroid for temperature sensing in the high-temperature regime, whose resolution, i.e. δT is much better there, improving from around 3 K to *ca.* 1 K at 800 K. Please note, that the final sensing accuracy of the temperature readouts with the proposed optical methods is affected by the wavelength resolution of the external spectroscopic equipment used, the number of measurements and acquisition time, signal-to-noise ratio (related to the luminescence performance and sensitivity of the optical detection setup used), as well as the validity of the data processing methods used, including curve fitting and spectral deconvolution.

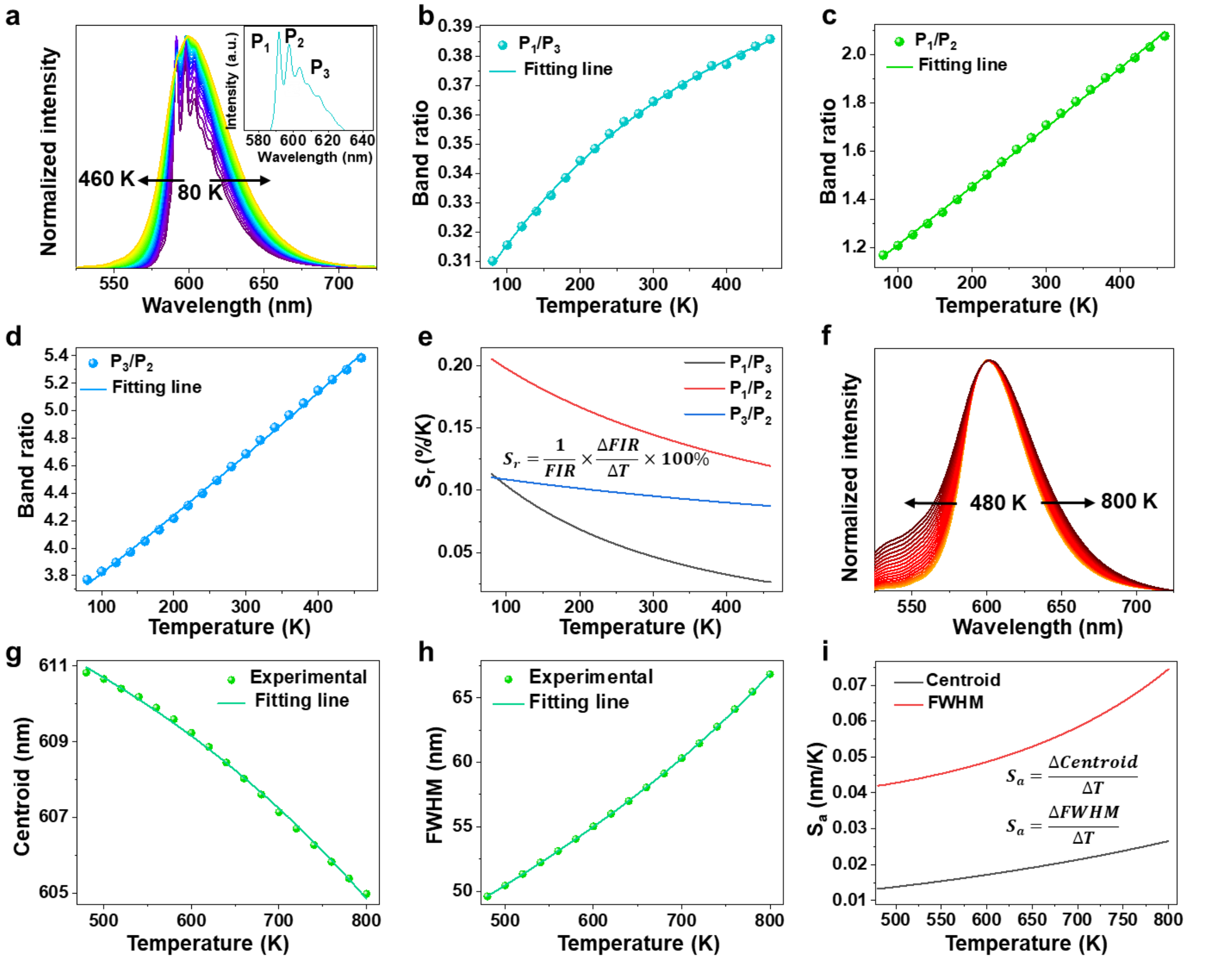

Fig. S7. The temperature sensing properties. (a) The PL emission spectra measured from 80 K to 460 K. The inset shows the marked peak 1 (P1), peak 2 (P2) and peak 3 (P3). (b-d) the corresponding emission band intensity ratio as a function of temperature. (e) The corresponding relative sensitivity (S_r) as a function of temperature. (f) The PL emission spectra measured from 480 K to 800 K. (g-h) The temperature dependence of emission band centroid and FWHM. (i) The corresponding absolute sensitivity (S_a) as a function of temperature.

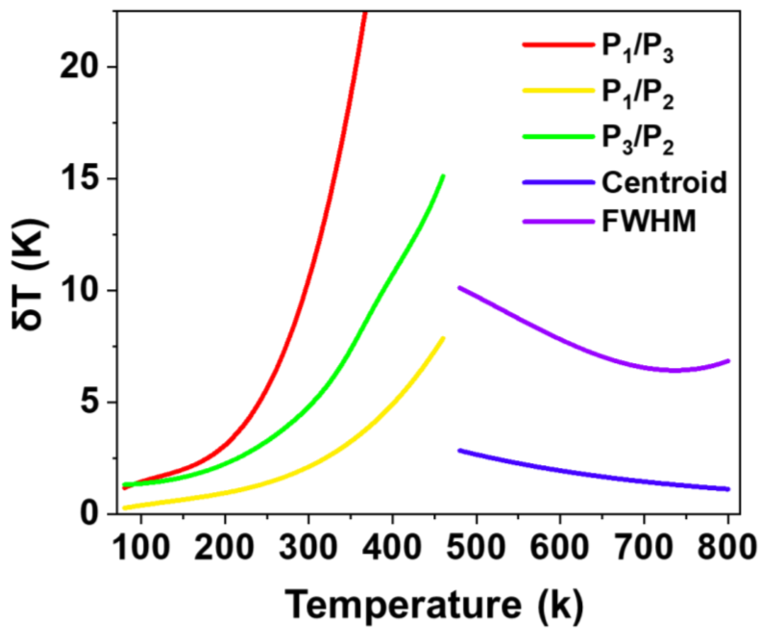

Fig. S8. The determined temperature sensing resolution – δT (uncertainty) for all thermometric parameters used, i.e. for three different band intensity ratios, emission band centroid and FWHM.

Supplementary Text S3: Trap depth distribution and charge dynamics

In order to study the traps contributing to the persistent luminescence, the initial rise method was applied (Fig. S9). First, the AlN:Mn^{2+} sample was excited during 1 minute at 100K. Then, a thermal cleaning (heating rate of 2K/s) was performed until a set temperature. Next, the sample was cooled again to 100K, before the full TL read-out from 100 to 500K occurred. From the slope of the initial part of the glow curve (until 10% of the maximum TL intensity), the corresponding trap depth energy could be calculated, by the so-called initial rise method. The experiment was repeated for thermal cleaning up to 110K, 120K and then in steps of 20K to 440K.

The trap depth energies form a continuous distribution ranging from 0.20 to 1.05 eV, rather than a limited number of discrete levels. The high density of shallow traps accounts for the persistent luminescence observed after excitation at 100 K.

To assess the contribution of tunnelling and trap-to-trap hopping, a series of five TL experiments was performed after filling the traps by UV-C excitation at 100 K (Fig. S10). In the first experiment, the TL glow curve (heating rate of 2 K/s) was recorded immediately after excitation, whereas in the second experiment the TL read-out was delayed by 10 minutes.

Comparing both glow curves shows that the shallowest traps are fully or partially emptied. In addition, a reduction of the TL intensity is observed up to ~320 K, although for the corresponding trap depths no significant thermal release is expected at 100 K on a 10 min timescale. This indicates the presence of tunnelling-assisted charge release in addition to thermally activated processes.

To further probe this effect, additional experiments were performed in which the sample was thermally cleaned up to 200 K and subsequently cooled back to 100 K. After this treatment, persistent luminescence was still observed. Subsequently, full TL glow curves up to 500 K were recorded either immediately or after delays of 10 and 20 minutes. As expected, the glow curves start around 200 K due to the removal of shallower traps during thermal cleaning. However, an additional delay at 100 K partially depletes traps that normally empty only between 200 and 350 K, providing further evidence for tunnelling-assisted charge release. Moreover, an increase in TL intensity between 160 and 200 K is observed for longer delay times, indicating charge redistribution between traps with different depths even at 100 K.

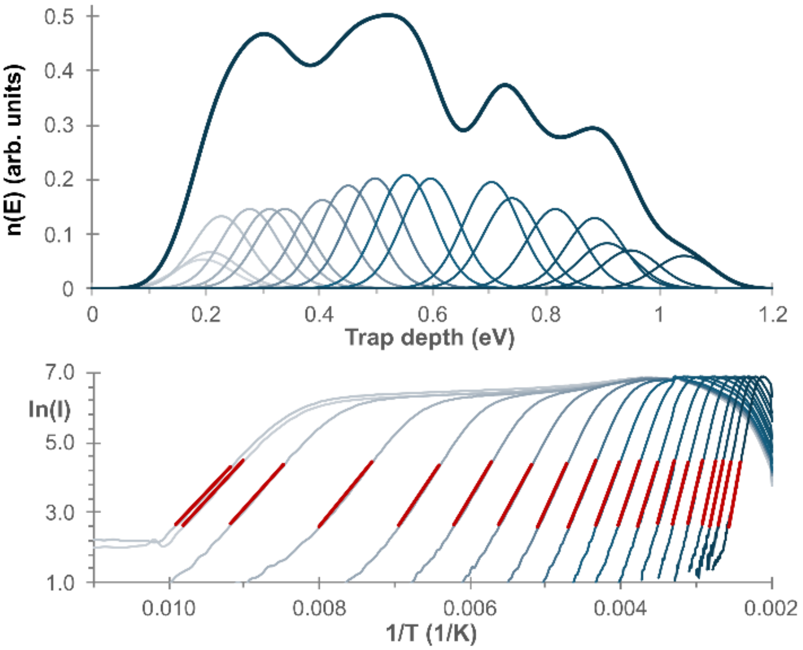

Fig. S9. Initial rise analysis after excitation at 100 K. TL glow curves recorded after partial thermal cleaning to temperatures between 110 and 440 K were used to extract trap depths from the initial part of each curve. The resulting trap depth distribution was reconstructed by convolution with narrow Gaussian functions.

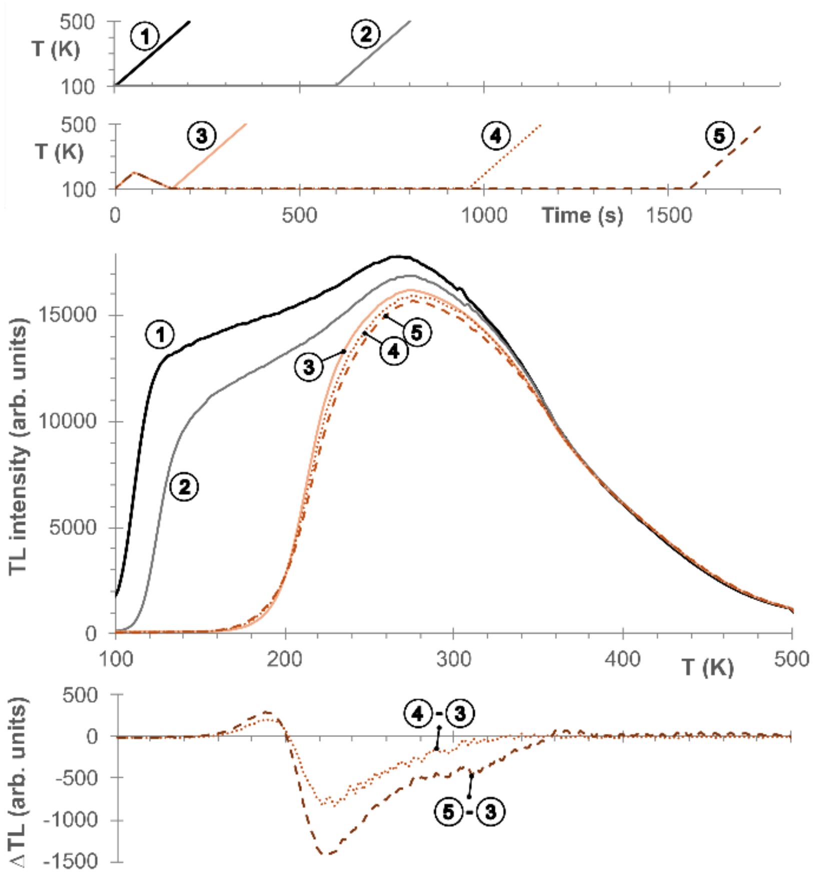

Fig. S10. (top) Temperature profiles used, after excitation with UV-C at 100K. (middle) Corresponding TL glow curves. (bottom) Difference plot between the glow curves found in experiments 4 and 3, and between experiments 5 and 3.

Supplementary Text S4: F-ML measurements

This paragraph refers to the results of F-ML measurements shown in Fig. 2c. For F-ML measurement, a glass rod was pressed against the sample plate with preset forces ranging from 0 N to 25 N and dragged across the plate at a speed of 6 mm/s, four times every 10 seconds. Each measurement, the sample was irradiated with 280 nm light for 2 minutes. For each force value, the rod started its first of four movements 30 s after the end of the sample irradiation stage. The emission spectra were collected every 60 ms. Since AlN:Mn exhibits very strong PersL at room temperature, the sample plate was kept in the dark before each F-ML experiment until the PersL signal faded enough, reaching no more than 7 counts/60 ms above the dark background (1 ± 2 counts/60 ms). Next, the sample was exposed to 280 nm from the LED for 2 minutes, and a subsequent F-ML experiment was started.

Supplementary Text S5: ML performance of AlN vs. AlN:Mn^{2+}

Fig. S11a shows comparison of the F-ML spectra for the AlN:Mn^{2+} and the pure AlN material at 30 N, indicating that the ML of the host is blue-shifted and much less intense compared to the Mn^{2+}-doped material studied in this work. Additionally, Fig. S11b quantitatively compared the evolution of the integrated ML intensity for both materials as a function of force, ranging from 5 to 30 N, and conforming that the Mn^{2+}-doping significantly enhances the ML intensity of the final material in the whole measre force range, compared to the ML of the pure AlN host. More details about the mechanisms governing the intrinsic ML from the pure AlN host can be found in Ref. (*61*).

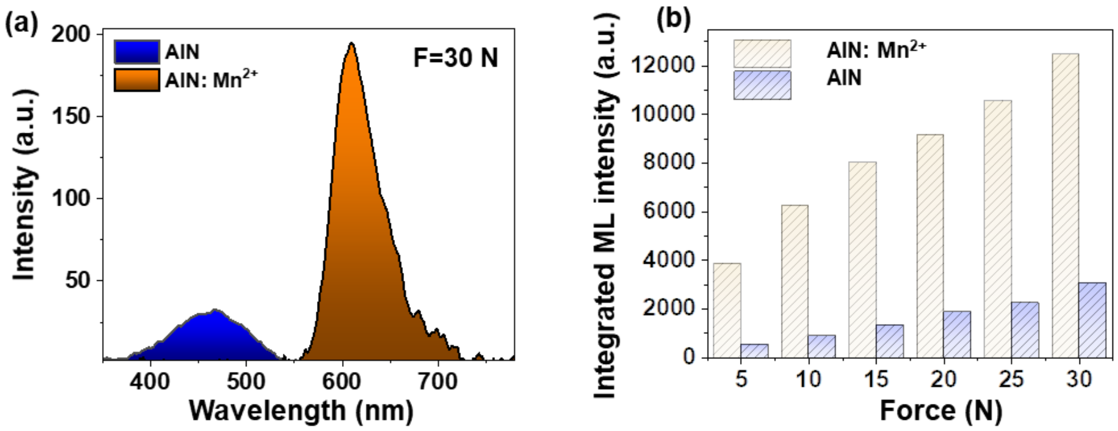

Fig. S11. (a) Comparison of the F-ML spectra for the AlN:Mn^{2+} material with pure AlN at 30 N. (b) Comparison of integrated F-ML intensity for the pure and Mn^{2+}-doped AlN materials as a function of force (5-30 N).

Supplementary Text S6: Refilling of the ML-active traps after the emission event

Note, that a purely static picture of trap depletion during mechanoluminescence (ML) would imply progressive exhaustion of stored charge, which is not consistent with the experimentally observed repeatability of the ML response. This indicates that the ML process in AlN:Mn must be understood as a dynamic, non-equilibrium phenomenon involving coupled detrapping and re-trapping processes.

In the wurtzite AlN host, mechanical stimulation generates local piezoelectric fields that not only trigger the release of carriers from mechanically sensitive traps but also drive carrier migration and subsequent re-trapping at other defect sites. As a result, ML does not correspond to a simple emptying of traps, but rather to a redistribution of carriers within a broad trap landscape. Importantly, detrapping and re-trapping can occur concurrently under mechanical perturbation. In addition, the persistent luminescence behavior evidences the presence of a wide distribution of trap depths, including deep traps that act as a long-lived reservoir of charge carriers. Slow thermally assisted transfer from these deeper states can continuously replenish shallower, ML-active traps, even in the absence of external excitation. Taken together, these processes establish a dynamic quasi-equilibrium of trapped carriers, in which the population relevant for ML is sustained over multiple excitation cycles. In this context, the "refilling" of traps is not a discrete process, but an emergent consequence of piezoelectrically driven carrier redistribution and thermally mediated trap coupling within a metastable charge configuration.

Supplementary Text S7: Piezoelectric performance

Please note that conventional piezoelectrics (e.g., PZT, $BaTiO_3$) are ferroelectric materials (*62*, *63*). Such materials contain numerous electrical domains with randomly oriented spontaneous polarization. A strong DC electric field (poling) aligns these domains, creating a net remanent polarization. This macroscopic alignment is essential for their strong piezoelectric response, and d_{33} measures this collective effect (*64*). In contrast to ferroelectric piezoelectric materials, AlN is a non-ferroelectric piezoelectric material whose response originates intrinsically from its non-centrosymmetric wurtzite crystal structure. AlN lacks switchable electrical domains and does not require a poling process to align dipoles, as the polarization direction of each grain is permanently determined during crystallization by the orientation of its crystallographic c-axis (*48*, *65*).

Consequently, the AlN:Mn^{2+} polycrystalline powder examined in this study exhibited no measurable piezoelectric response, which is consistent with the behavior of unpoled, non-ferroelectric polycrystalline materials where random crystallographic orientations lead to cancellation of individual grain contributions at the macroscopic scale. In order to measure the piezoelectric properties of the studied AlN material, the value of piezoelectric strain constant d_{33} for the single crystal of the pure AlN matrix, is measured by a quasi-static piezoelectric meter (ZJ-4AN, CAS, Shanghai, China). The synthesize process can be found in our previous paper (*61*). When piezoelectric materials are subjected to an external force along the polarization direction, charges proportional to the magnitude of the external force will be generated on the two electrode surfaces of the material. The d_{33} value, i.e. the piezoelectric coefficient of the material, is calculated

by the formula $d_{33} = \Delta Q/(\Delta F/A)$ (where ΔQ is the change in charge, ΔF is the change in external force, and A is the electrode area). Here, we obtained the $d_{33} \approx 4.6$ PC/N for undoped AlN single crystal. Such a result is close to previous report. For example, Kim et al reported a d_{33} of 6.5 pC/N for AlN single crystal, and a increased value of 13.5 pC/N at 1000 ℃ (*46*). Matin et al reported a d_{33} of 5.15 pC/N for highly c-axis textured AlN thin films (*47*). This is due to that AlN single crystal exhibits a single-domain structure without domain walls or phase boundaries, which eliminates extrinsic contributions to piezoelectricity that are often exploited in ferroelectric ceramics to achieve high d_{33} coefficients (*48*, *62*, *65*, *66*). The piezoelectric behavior in AlN is thus purely intrinsic, arising from the relative displacement of aluminum and nitrogen ions within the crystal lattice under mechanical or electrical stress. This displacement, however, is constrained by the strong covalent bonding characteristic of the Al–N bonds in the wurtzite structure, inherently limiting the magnitude of its piezoelectric coefficients compared to ferroelectric materials.

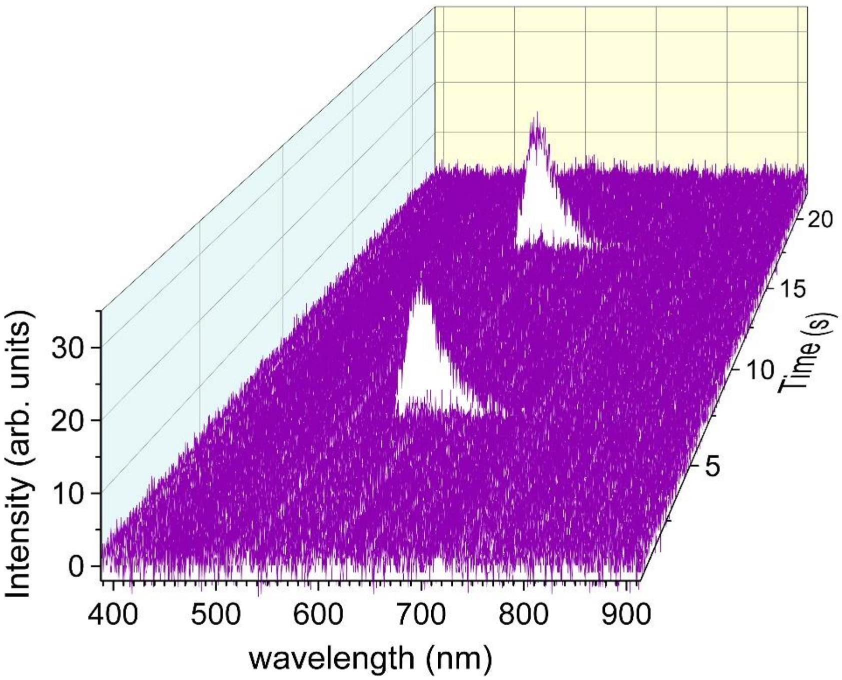

Fig. S12. F-ML spectra of the AlN:Mn^{2+}, both measured under the same conditions, i.e. under a 0.2 N load (~8 bar; 0.8 MPa) applied by a glass rod, scanned at 36 mm/s (two passes; 10 s interval), and recorded after keeping the material one week in darkness.

Supplementary Text S8: Ultrasound-induced mechanoluminescence

Two experimental setups and multiple approaches were employed to test US-ML at ultrasound frequencies of 20 kHz and 3.3 MHz. Before each US-ML experiment, the sample sheet was irradiated with 270 nm from an LED for 1 minute and then kept in the dark for an additional minute, after which the sample was exposed to an ultrasonic beam, with parameters depending on the type of experiment being conducted (described in more detail below). To validate the reproducibility of US-ML, we measured US-ML over several cycles of sample sheet exposure to the ultrasonic beam without UV recharging between cycles.

Fig. S6 depicts the US-ML intensity measured at ultrasound frequency of 20 kHz for different US waves on/off time ratios (1 s/19 s, 2 s/18 s, 3 s/17 s, 4 s/16 s, 5 s/15 s, and 8 s/12 s), applied across five cycles and at 24 μm vibrational amplitude. The US-ML peaks decrease with the number of pulses, and saturation of each peak occurs at higher on/off ratios and with an increasing number of pulses. Fig. S6b shows the peak US-ML intensity as a function of the on-duration of each US pulse, calculated as the ultrasound-induced increase in the intensity of light emitted by the sample, relative to the decaying PersL signal taken without ultrasound stimulation. Fig. S6c shows the total US-ML intensity as a function of the on-duration of each US pulse, calculated by integrating the US-ML signals over time. The corresponding camera frames are shown above the symbols representing the measured US-ML intensities. The variations in US-ML intensities at different points of a frame are reflected in the color scale.

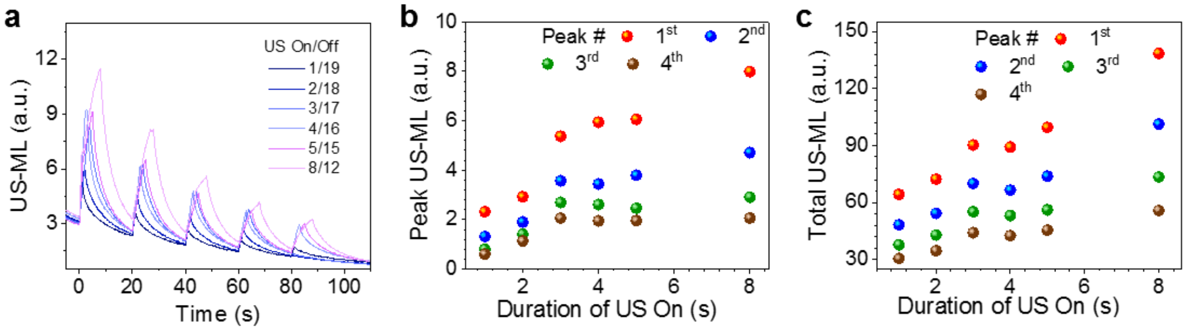

Fig. S13: (a) Intensity of US-ML as a function of time at 24 µm vibration amplitude and different On/Off ratio 1 s/19 s, 2 s/18 s, 3 s/17 s, 4 s/16 s, 5 s/15 s and 8 s/12 s. (b) Peak US-L values for 1st, 2nd, 3rd and 4th US-Ml peak vs. duration of US exposure. (c) Total US-ML values for 1st, 2nd, 3rd and 4th peak vs. duration of US exposure.

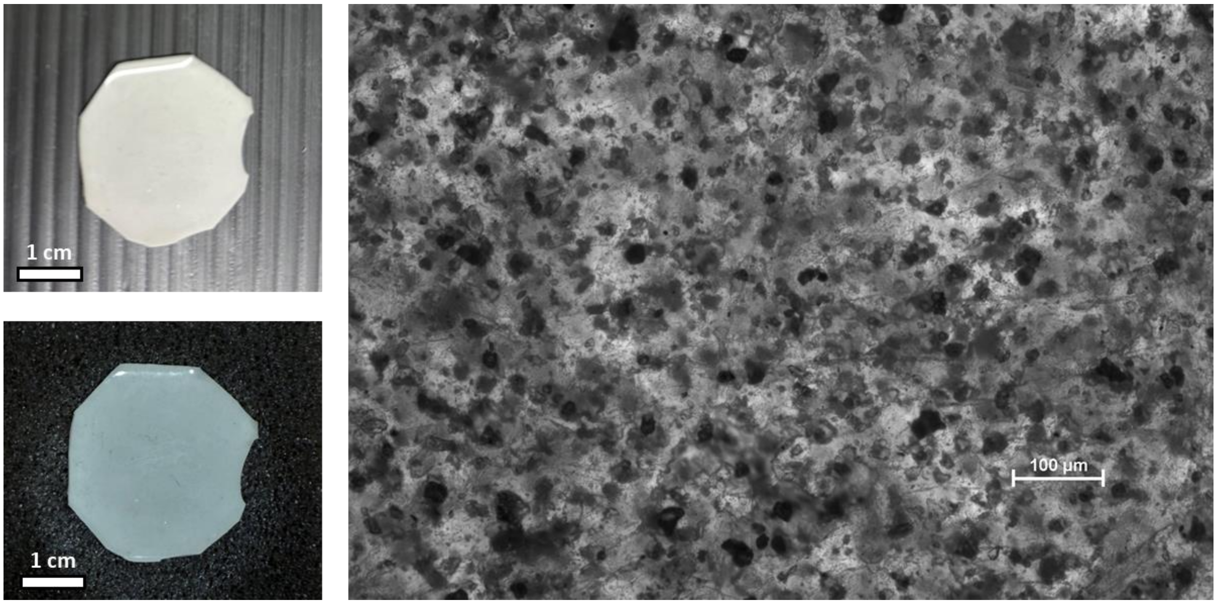

Fig. S14. Photographs taken in a daylight under different illumination conditions (left) and the corresponding micrograph taken with the fluorescence microscope under white light illumination (right) for the AlN:Mn^{2+}-modified PDMS film.

Table S1. The fitting parameters in the thermometric analysis were obtained using the fitting function: $y = A_1 exp\left(-\frac{T}{t_1}\right) + A_2 exp\left(-\frac{T}{t_2}\right) + y_0$, where y represents the band ratio, band centroid, or FWHM.

	A_1	t_1	A_2	t_2	y_0	R^2
**Band ratio P_1/P_3**	-0.069	306.134	-0.070	306.153	*0.416*	0.999
**Band ratio P_1/P_2**	9.789	-8289.462	9.795	8264.066	*-18.611*	0.999
**Band ratio P_3/P_2**	5.462	-2738.84	5.480	-2735.11	*-7.533*	0.998
**Centroid**	-1.036	-458.61	-1.091	-458.522	*617.025*	0.998
**FWHM**	*0.137*	*-196.888*	*7094.881*	*-209446.292*	*-7063.120*	0.999

Movie S1. Long-lasting afterglow emission of the AlN:Mn^{2+}-modified PDMS film.
Movie S2. Thermoluminescence of the AlN:Mn^{2+}-modified PDMS film.
Movie S3. Mechanoluminescence (via handwriting) of the AlN:Mn^{2+}-modified PDMS film.
Movie S4. Mechanoluminescence (via bending) of the AlN:Mn^{2+}-modified PDMS film.
Movie S5. Mechanoluminescence (via stretching) of the AlN:Mn^{2+}-modified PDMS film.
Movie S6. Mechanoluminescence (via ultrasounds) of the AlN:Mn^{2+}-modified PDMS film.

REFERENCES

1. J. R. Casar, C. A. McLellan, C. Shi, A. Stiber, A. Lay, C. Siefe, A. Parakh, M. Gaerlan, X. W. Gu, M. B. Goodman, J. A. Dionne, Upconverting microgauges reveal intraluminal force dynamics in vivo. *Nature* **637**, 76–83 (2025).

2. Y. Zhuang, R.-J. Xie, Mechanoluminescence rebrightening the prospects of stress sensing: A review. *Adv. Mater.* **33**, e2005925 (2021).

3. X. Y. Wei, X. Wang, S. Y. Kuang, L. Su, H. Y. Li, Y. Wang, C. Pan, Z. L. Wang, G. Zhu, Dynamic triboelectrification-induced electroluminescence and its use in visualized sensing. *Adv. Mater.* **28**, 6656–6664 (2016).

4. W. Li, Y. Cai, J. Chang, J. Liu, S. Wang, J.-C. Zhang, Exploring mechanoluminescence of zinc alkaline earth metal oxysulfides from fundamentals to advanced applications. *Adv. Funct. Mater.* **35**, 2412494 (2025).

5. A. Qasem, P. Xiong, Z. Ma, M. Peng, Z. Yang, Recent advances in mechanoluminescence of doped zinc sulfides. *Laser Photonics Rev.* **15**, 2100276 (2021).

6. Y. Ding, B. So, J. Cao, L. Wondraczek, Ultrasound-induced mechanoluminescence and optical thermometry toward stimulus-responsive materials with simultaneous trigger response and read-out functions. *Adv. Sci.* **9**, e2201631 (2022).

7. T. G. Leighton, What is ultrasound? *Prog. Biophys. Mol. Biol.* **93**, 3–83 (2007).

8. P. Xiong, M. Peng, J. Cao, X. Li, Near infrared mechanoluminescence from $Sr_3 Sn_2 O_7$: Nd^{3+} for in situ biomechanical sensor and dynamic pressure mapping. *J. Am. Ceram. Soc.* **102**, 5899–5909 (2019).

9. Y. Zhang, Y. Fang, J. Li, Q. Zhou, Y. Xiao, K. Zhang, B. Luo, J. Zhou, B. Hu, Dual-mode electronic skin with integrated tactile sensing and visualized injury warning. *ACS Appl. Mater. Interfaces* **9**, 37493–37500 (2017).

10. X. Qian, Z. Cai, M. Su, F. Li, W. Fang, Y. Li, X. Zhou, Q. Li, X. Feng, W. Li, X. Hu, X. Wang, C. Pan, Y. Song, Printable skin-driven mechanoluminescence devices via nanodoped matrix modification. *Adv. Mater.* **30**, e1800291 (2018).

11. H. Li, Y. Zhang, H. Dai, W. Tong, Y. Zhou, J. Zhao, Q. An, A self-powered porous ZnS/PVDF-HFP mechanoluminescent composite film that converts human movement into eye-readable light. *Nanoscale* **10**, 5489–5495 (2018).

12. R. R. Petit, S. E. Michels, A. Feng, P. F. Smet, Adding memory to pressure-sensitive phosphors. *Light Sci. Appl.* **8**, 124 (2019).

13. W. Wu, Z. L. Wang, Piezotronics and piezo-phototronics for adaptive electronics and optoelectronics. *Nat. Rev. Mater.* **1**, 16031 (2016).

14. C. Pan, L. Dong, G. Zhu, S. Niu, R. Yu, Q. Yang, Y. Liu, Z. L. Wang, High-resolution electroluminescent imaging of pressure distribution using a piezoelectric nanowire LED array. *Nat. Photonics* **7**, 752–758 (2013).

15. K. Chen, X. Yuan, Z. Tian, M. Zou, Y. Yuan, Z. Chen, Q. Zhang, Y. Zhang, X. Jin, T. Wu, R. Shahbazian-Yassar, G. Liu, A facile approach for generating ordered oxygen vacancies in metal oxides. *Nat. Mater.* **24**, 835–842 (2025).

16. Y. Taniyasu, M. Kasu, T. Makimoto, An aluminium nitride light-emitting diode with a wavelength of 210 nanometres. *Nature* **441**, 325–328 (2006).

17. H. Chang, Z. Liu, S. Yang, Y. Gao, J. Shan, B. Liu, J. Sun, Z. Chen, J. Yan, Z. Liu, J. Wang, P. Gao, J. Li, Z. Liu, T. Wei, Graphene-driving strain engineering to enable strain-free epitaxy of AlN film for deep ultraviolet light-emitting diode. *Light Sci. Appl.* **11**, 88 (2022).

18. X. Hao, S. Wan, S. Du, Z. Chen, J. Zhang, X. Yuan, Q. Wang, Synthesis and luminescence properties of red-emitting AlN:Mn^{2+} spherical phosphors. *J. Am. Ceram. Soc.* **105**, 6787–6798 (2022).

19. L. Yang, H. Zou, X. Wang, C. Wang, Q. Wang, C. Liu, Synthesis, magnetism and photoluminescence of Mn doped AlN nanowires. *J. Lumin.* **243**, 118657 (2022).

20. Y. Zhao, G. Bai, Y. Huang, Y. Liu, D. Peng, L. Chen, S. Xu, Stimuli responsive lanthanide ions doped layered piezophotonic microcrystals for optical multifunctional sensing applications. *Nano Energy* **87**, 106177 (2021).

21. N. J. Cherepy, S. A. Payne, N. M. Harvey, D. Åberg, Z. M. Seeley, K. S. Holliday, I. C. Tran, F. Zhou, H. P. Martinez, J. M. Demeyer, A. D. Drobshoff, A. M. Srivastava, S. J. Camardello, H. A. Comanzo, D. L. Schlagel, T. A. Lograsso, Red-emitting manganese-doped aluminum nitride phosphor. *Opt. Mater.* **54**, 14–21 (2016).

22. A. Hung, S. P. Russo, D. G. McCulloch, S. Prawer, An ab initio study of structural properties and single vacancy defects in wurtzite AlN. *J. Chem. Phys.* **120**, 4890–4896 (2004).

23. C. G. Van De Walle, J. Neugebauer, First-principles calculations for defects and impurities: Applications to III-nitrides. *J. Appl. Phys.* **95**, 3851–3879 (2004).

24. Y. G. Cao, X. L. Chen, Y. C. Lan, J. Y. Li, Y. P. Xu, T. Xu, Q. L. Liu, J. K. Liang, Blue emission and Raman scattering spectrum from AlN nanocrystalline powders. *J. Cryst. Growth* **213**, 198–202 (2000).

25. Q. Dong, Y. Yao, S. Cheng, K. Alexopoulos, J. Gao, S. Srinivas, Y. Wang, Y. Pei, C. Zheng, A. H. Brozena, H. Zhao, X. Wang, H. E. Toraman, B. Yang, I. G. Kevrekidis, Y. Ju, D. G. Vlachos, D. Liu, L. Hu, Programmable heating and quenching for efficient thermochemical synthesis. *Nature* **605**, 470–476 (2022).

26. M. Liu, Q. Wan, H. Wang, F. Carulli, X. Sun, W. Zheng, L. Kong, Q. Zhang, C. Zhang, Q. Zhang, S. Brovelli, L. Li, Suppression of temperature quenching in perovskite nanocrystals for efficient and thermally stable light-emitting diodes. *Nat. Photonics* **15**, 379–385 (2021).

27. L. Wu, S. Sun, Y. Bai, Z. Xia, L. Wu, H. Chen, L. Zheng, H. Yi, T. Sun, Y. Kong, Y. Zhang, J. Xu, Defect-induced self-reduction and anti-thermal quenching in $NaZn(PO_3)_3:Mn^{2+}$ red phosphor. *Adv. Opt. Mater.* **9**, 2100870 (2021).

28. V. Fritz, D. Van Der Heggen, P. F. Smet, Validated approach to measure thermal quenching in the presence of trapping defects. *ACS Photonics* **11**, 1981–1989 (2024).

29. H.-Y. Kai, K.-L. Wong, P. A. Tanner, Tunneling in persistent luminescence. *Adv. Opt. Mater.* **13**, 2500793 (2025).

30. A. Dobrowolska, A. J. J. Bos, P. Dorenbos, Electron tunnelling phenomena in YPO_4: Ce,Ln (Ln = Er, Ho, Nd, Dy). *J. Phys. D Appl. Phys.* **47**, 335301 (2014).

31. H. Liu, Y. Zheng, S. Liu, J. Zhao, Z. Song, D. Peng, Q. Liu, Realizing red mechanoluminescence of ZnS: Mn^{2+} through ferromagnetic coupling. *Adv. Funct. Mater.* **34**, 2314422 (2024).

32. J.-C. Zhang, L.-Z. Zhao, Y.-Z. Long, H.-D. Zhang, B. Sun, W.-P. Han, X. Yan, X. Wang, Color manipulation of intense multiluminescence from CaZnOS:Mn^{2+} by Mn^{2+} concentration effect. *Chem. Mater.* **27**, 7481–7489 (2015).

33. H. Liu, Y. Shao, C. Dou, J. Zhao, Z. Song, Q. Liu, Tuning mechanoluminescence from red to near-infrared light in CaZnOS:Mn^{2+}. *Adv. Opt. Mater.* **13**, 2403472 (2025).

34. M. Vening, D. J. Dunstan, K. P. Homewood, Thermal quenching and retrapping effects in the photoluminescence of $In_yGa_{1-y}As/GaAs/Al_xGa_{1-x}As$ multiple-quantum-well structures. *Phys. Rev. B* **48**, 2412–2417 (1993).

35. N. G. Debelo, F. B. Dejene, V. N. Mal'nev, T. Senbeta, B. Mesfin, K. Roro, Effect of retrapping on thermoluminescence peak intensities of small amorphous silicon quantum dots. *Acta Phys. Pol. A* **129**, 362–366 (2016).

36. A. Lazarowska, M. Kamiński, N. J. Cherepy, S. Mahlik, R.-S. Liu, Dual role of oxygen-related defects in the luminescence kinetics of AlN:Mn^{2+}. *Dalton Trans.* **51**, 14297–14305 (2022).

37. A. I. Ryskin, A. S. Shcheulin, B. Koziarska, J. M. Langer, A. Suchocki, I. I. Buczinskaya, P. P. Fedorov, B. P. Sobolev, CdF2:In: A novel material for optically written storage of information. *Appl. Phys. Lett.* **67**, 31–33 (1995).

38. J. Nissilä, K. Saarinen, P. Hautojärvi, A. Suchocki, J. M. Langer, Universality of the bond-breaking mechanism in defect bistability: Observation of open volume in the deep states of In and Ga in CdF_2. *Phys. Rev. Lett.* **82**, 3276–3279 (1999).

39. S. B. Orlinskii, J. Schmidt, P. G. Baranov, M. Bickermann, B. M. Epelbaum, A. Winnacker, Observation of the triplet metastable state of shallow donor pairs in AlN crystals with a negative-U behavior: A high-frequency EPR and ENDOR study. *Phys. Rev. Lett.* **100**, 256404 (2008).

40. M. Hayden Breckenridge, Q. Guo, A. Klump, B. Sarkar, Y. Guan, J. Tweedie, R. Kirste, S. Mita, P. Reddy, R. Collazo, Z. Sitar, Shallow Si donor in ion-implanted homoepitaxial AlN. *Appl. Phys. Lett.* **116**, 172103 (2020).

41. N. T. Son, M. Bickermann, E. Janzén, Shallow donor and *DX* states of Si in AlN. *Appl. Phys. Lett.* **98**, 092104 (2011).

42. J. Xu, N. J. Cherepy, J. Ueda, S. Tanabe, Red persistent luminescence in rare earth-free $AlN:Mn^{2+}$ phosphor. *Mater. Lett.* **206**, 175–177 (2017).

43. A. K. Somakumar, Y. Zhydachevskyy, D. Wlodarczyk, S. S. Haider, J. Barzowska, K. R. Bindu, Y. K. Edathumkandy, T. Zayarniuk, A. Szewczyk, S. Narayanan, A. Lysak, H. Przybylinska, E. I. Anila, A. Suchocki, Temperature and pressure dependent luminescence mechanism of a zinc blende structured ZnS:Mn nanophosphor under UV excitation. *J. Mater. Chem. C* **12**, 7041–7052 (2024).

44. A. K. Somakumar, L.-I. Bulyk, V. Tsiumra, J. Barzowska, P. Xiong, A. Lysak, Y. Zhydachevskyy, A. Suchocki, High-pressure near-infrared luminescence studies of Fe^{3+}-activated $LiGaO_2$. *Inorg. Chem.* **62**, 12434–12444 (2023).

45. Y.-T. Tsai, T. Leśniewski, N. Majewska, M. Kamiński, J. Barzowska, E.-P. Liu, W.-T. Chen, S. Mahlik, M.-H. Fang, Pressure/temperature-assisted crystallographic engineering–A strategy for developing the infrared phosphors. *Chem. Eng. J.* **490**, 151596 (2024).

46. T. Kim, J. Kim, R. Dalmau, R. Schlesser, E. Preble, X. Jiang, High-temperature electromechanical characterization of AlN single crystals. *IEEE Trans. Ultrason. Ferroelectr. Freq. Control* **62**, 1880–1887 (2015).

47. F. Martin, P. Muralt, M.-A. Dubois, A. Pezous, Thickness dependence of the properties of highly *c*-axis textured AlN thin films. *J. Vac. Sci. Technol. Vac. Surf. Films* **22**, 361–365 (2004).

48. H. Yang, J. Sun, H. Wang, H. Li, B. Yang, A review of oriented wurtzite-structure aluminum nitride films. *J. Alloys Compd.* **989**, 174330 (2024).

49. S. S. Haider, J. Barzowska, P. Sybilski, A. Suchocki, Designing of experimental setup for impact induced mechanoluminescence measurements. *Measurement* **203**, 112012 (2022).

50. S. E. Michels, M. Kersemans, M. Versluis, G. Lajoinie, P. F. Smet, Fast and high-resolution ultrasound pressure field mapping using luminescent membranes. *Adv. Opt. Mater.* **9**, 2100085 (2021).

51. X. Pan, Y. Zhuang, W. He, C. Lin, L. Mei, C. Chen, H. Xue, Z. Sun, C. Wang, D. Peng, Y. Zheng, C. Pan, L. Wang, R.-J. Xie, Quantifying the interfacial triboelectricity in inorganic-organic composite mechanoluminescent materials. *Nat. Commun.* **15**, 2673 (2024).

52. T. Zheng, M. Sójka, M. Runowski, P. Woźny, S. Lis, E. Zych, Tm^{2+} activated SrB_4O_7 bifunctional sensor of temperature and pressure—Highly sensitive, multi-parameter luminescence thermometry and manometry. *Adv. Opt. Mater.* **9**, 2101507 (2021).

53. S. Du, D. Wang, Q. Qiang, X. Ma, Z. Tang, Y. Wang, The dual-model up/down-conversion green luminescence of $Gd_6O_5F_8$:Yb^{3+},Ho^{3+},Li^+ and its application for temperature sensing. *J. Mater. Chem. C* **4**, 7148–7155 (2016).

54. C. D. S. Brites, A. Millán, L. D. Carlos, "Lanthanides in Luminescent Thermometry" in *Handbook on the Physics and Chemistry of Rare Earths* (Elsevier, 2016), vol. 49, pp. 339–427.

55. X. Zhang, Y. Huang, M. Gong, Dual-emitting Ce^{3+}, Tb^{3+} co-doped LaOBr phosphor: Luminescence, energy transfer and ratiometric temperature sensing. *Chem. Eng. J.* **307**, 291–299 (2017).

56. Y. Gao, F. Huang, H. Lin, J. Zhou, J. Xu, Y. Wang, A novel optical thermometry strategy based on diverse thermal response from two intervalence charge transfer states. *Adv. Funct. Mater.* **26**, 3139–3145 (2016).

57. D. K. Amarasinghe, F. A. Rabuffetti, Bandshift luminescence thermometry using Mn^{4+}:$Na_4Mg(WO_4)_3$ phosphors. *Chem. Mater.* **31**, 10197–10204 (2019).

58. W. Zhang, G. Wang, Z. Cai, G. W. Baxter, S. F. Collins, Spectral analysis for broadband fluorescence: Temperature sensing with the YAG:Ce phosphor as an example. *Opt. Mater. Express* **6**, 3482–3490 (2016).

59. M. A. Hernández-Rodríguez, A. D. Lozano-Gorrín, I. R. Martín, U. R. Rodríguez-Mendoza, V. Lavín, Comparison of the sensitivity as optical temperature sensor of nano-perovskite doped with Nd^{3+} ions in the first and second biological windows. *Sens. Actuators B Chem.* **255**, 970–976 (2018).

60. T. Zheng, L. Luo, P. Du, S. Lis, U. R. Rodríguez-Mendoza, V. Lavín, M. Runowski, Highly-efficient double perovskite Mn^{4+}-activated Gd_2ZnTiO_6 phosphors: A bifunctional optical sensing platform for luminescence thermometry and manometry. *Chem. Eng. J.* **446**, 136839 (2022).

61. T. Zheng, P. Woźny, K. Soler-Carracedo, D. Han, J. Wang, L. Peng, W. Li, D. Peng, H. Wu, J. Moszczyński, S. Mahlik, M. Runowski, Mechanoluminescent aluminum nitride crystal for super-sensitive optical manometry, thermometry and force sensing. *Adv. Mater.* **38**, e11943 (2026).

62. Z.-Y. Shen, J.-F. Li, Enhancement of piezoelectric constant d_{33} in $BaTiO_3$ ceramics due to nano-domain structure. *J. Cerma. Soc. Jpn.* **118**, 940–943 (2010).

63. P. K. Panda, B. Sahoo, PZT to lead free piezo ceramics: A review. *Ferroelectrics* **474**, 128–143 (2015).

64. F. Li, D. Lin, Z. Chen, Z. Cheng, J. Wang, C. Li, Z. Xu, Q. Huang, X. Liao, L.-Q. Chen, T. R. Shrout, S. Zhang, Ultrahigh piezoelectricity in ferroelectric ceramics by design. *Nat. Mater.* **17**, 349–354 (2018).

65. T. Nguyen, N. Adjeroud, S. Glinsek, Y. Fleming, J. Guillot, P. Grysan, J. Polesel-Maris, A film-texture driven piezoelectricity of AlN thin films grown at low temperatures by plasma-enhanced atomic layer deposition. *APL Mater.* **8**, 071101 (2020).

66. A. N. Cleland, M. Pophristic, I. Ferguson, Single-crystal aluminum nitride nanomechanical resonators. *Appl. Phys. Lett.* **79**, 2070–2072 (2001).
